# The Differential Mobilization of Histones H3.1 and H3.3 by Herpes Simplex Virus 1 Relates Histone Dynamics to the Assembly of Viral Chromatin

**DOI:** 10.1371/journal.ppat.1003695

**Published:** 2013-10-10

**Authors:** Kristen L. Conn, Michael J. Hendzel, Luis M. Schang

**Affiliations:** 1 Department of Biochemistry, University of Alberta, Edmonton, Alberta, Canada; 2 Department of Experimental Oncology, University of Alberta, Edmonton, Alberta, Canada; 3 Department of Medical Microbiology and Immunology, University of Alberta, Edmonton, Alberta, Canada; University of Wisconsin-Madison, United States of America

## Abstract

During lytic infections, HSV-1 genomes are assembled into unstable nucleosomes. The histones required for HSV-1 chromatin assembly, however, are in the cellular chromatin. We have shown that linker (H1) and core (H2B and H4) histones are mobilized during HSV-1 infection, and proposed that the mobilized histones are available for assembly into viral chromatin. However, the actual relevance of histone mobilization remained unknown. We now show that canonical H3.1 and variant H3.3 are also mobilized during HSV-1 infection. Mobilization required no HSV-1 protein expression, although immediate early or early proteins enhanced it. We used the previously known differential association of H3.3 and H3.1 with HSV-1 DNA to test the relevance of histone mobilization. H3.3 binds to HSV-1 genomes first, whereas H3.1 only binds after HSV-1 DNA replication initiates. Consistently, H3.3 and H3.1 were differentially mobilized. H3.1 mobilization decreased with HSV-1 DNA replication, whereas H3.3 mobilization was largely unaffected by it. These results support a model in which previously mobilized H3.1 is immobilized by assembly into viral chromatin during HSV-1 DNA replication, whereas H3.3 is mobilized and assembled into HSV-1 chromatin throughout infection. The differential mobilizations of H3.3 and H3.1 are consistent with their differential assembly into viral chromatin. These data therefore relate nuclear histone dynamics to the composition of viral chromatin and provide the first evidence that histone mobilization relates to viral chromatin assembly.

## Author Summary

H3.1 is typically assembled into chromatin during DNA replication-dependent chromatin assembly. However, histones undergo exchange with those not bound in chromatin. During such exchanges, DNA replication-independent chromatin assembly incorporates histone variants, such as H3.3. The HSV-1 genomes are chromatinized, albeit in unstable nucleosomes. The viral genomes initially associate with H3.3, then associate with H3.1 only after HSV-1 DNA replication initiates. These differential interactions are consistent with the DNA replication-independent or -dependent assembly of H3.3 or H3.1, respectively, in cellular chromatin. We have shown that linker (H1) and core (H2B and H4) histones are mobilized during HSV-1 infection, but the significance of this mobilization remained unknown. We now find that H3.3 and H3.1 are also mobilized during infection. H3.3 is mobilized to a similar extent before or after HSV-1 DNA replication, which is consistent with its DNA replication-independent assembly into HSV-1 chromatin. In contrast, H3.1 mobilization decreases during HSV-1 DNA replication, which is consistent with the assembly of previously mobilized H3.1 into HSV-1 chromatin concomitant with HSV-1 DNA replication. The mobilizations of H3.1 and H3.3 are consistent with their kinetics of association with HSV-1 genomes, providing the first indication that histone mobilization relates to the assembly of viral chromatin.

## Introduction

Cellular DNA is wrapped around protein octamers containing two molecules each of histones H2A, H2B, H3, and H4, forming the nucleosome [1]. Linker histone H1 binds to DNA at the entry and exit sites of the core nucleosome to promote the formation of higher-order chromatin structures [2]. Nucleosomes are partially or completely disassembled to allow access to the DNA, and are subsequently re-assembled to reform the chromatin structure [3]. Chromatin thus physically modulates access to the DNA, regulating processes that require such access (e.g. gene expression, DNA replication, and DNA repair) [3]. The stability of the interactions between the histones within the nucleosome, between nucleosomes, and between nucleosomes and DNA, affects the stability and structure of chromatin, regulating access to the DNA [4]–[6].

The histone variants within the nucleosome affect the stability of the octamer and its associations with DNA [7], [8]. Canonical core histone H3.1 differs from the variant histone H3.3 in only four residues. These differences suffice to alter nucleosome interactions, such that nucleosomes containing H3.3 are less stable than those containing H3.1 [8]. They also dictate specific interactions with histone chaperones, which in turn mediate nucleosome assembly and disassembly. H3.1, which is expressed only during S-phase, specifically interacts with chromatin assembly factor 1 (CAF-1) and is deposited onto DNA primarily during DNA replication [9]. In contrast, H3.3, which is expressed throughout the cell cycle, specifically interacts with histone chaperone complexes containing histone regulator A (HIRA), hDaxx, or DEK [9]–[12]. Of them, HIRA mediates the assembly of H3.3 into nucleosomes within the transcription start sites (TSS) of active or repressed genes, and within the coding region of active genes, whereas hDaxx mediates its assembly into telomeric chromatin [13].

Gene expression of nuclear replicating dsDNA viruses, such as herpes simplex virus 1 (HSV-1), is epigenetically regulated (reviewed in [14]). HSV-1 genomes are tightly chromatinized and largely transcriptionally silent during latency, whereas they are assembled into unstable nucleosomes and abundantly transcribed during lytic infections [15]–[17]. Infecting HSV-1 genomes enter the nucleus bound by spermine, which is then replaced with histones [18], [19]. Later in infection, HSV-1 DNA replication produces additional HSV-1 genomes, which are also assembled into chromatin [17], [20]. Given that histone synthesis is inhibited during infection [21]–[23], the histones that are assembled into HSV-1 chromatin are not synthesized de novo. Therefore, the histones assembled into viral chromatin were assembled in, and undergoing exchange with, cellular chromatin before infection. The chromatin exchange of linker histone H1 and core histones H2B and H4 increases during HSV-1 infection [24], [25]. This mobilization of histones during lytic infections was proposed to provide a source of histones for the assembly of viral chromatin. However, the actual significance of histone mobilization remained unknown.

The deposition of specific histone variants onto cellular DNA is governed by histone chaperone complexes, which are coupled to specific chromatin assembly and disassembly pathways [26]. Although the assembly of HSV-1 chromatin has yet to be fully characterized, the differential deposition of H3.3 and H3.1 into cellular nucleosomes appears to be paralleled for HSV-1 nucleosomes. Whereas H3.3 is initially assembled into HSV-1 nucleosomes, H3.1 is only assembled into the viral chromatin when HSV-1 DNA replicates [27]. This differential association of H3.3 and H3.1 with HSV-1 genomes can be used to test whether the mobilization of histones is related to their assembly into HSV-1 chromatin.

The HSV-1 transcription activators VP16 and ICP0 induce chromatin remodeling and interact with a variety of cellular proteins that introduce histone post-translational modifications [28]–[37]. Both proteins modulate the mobilization of linker histone H1 and core histones H2B and H4 [24], [25]. Mutants in VP16 and ICP0 are deficient in HSV-1 gene expression and replication in the vast majority of cell lines, such as Vero cells [38]. However, the activities of these proteins are far less important in a particular cell line, U2OS [39], in which HSV-1 mutants in VP16 or ICP0 transcribe their genes and replicate with close to wild-type kinetics. To date, the mechanisms whereby U2OS cells complement such mutants remain mostly unknown. U2OS cells therefore provide an excellent model to test whether chromatin dynamics play a major role in cellular antiviral silencing that is counteracted by viral proteins such as ICP0 and VP16.

Here we report that H3.3 and H3.1 are mobilized during infection. H3.3 mobilization does not require HSV-1 proteins and is not drastically affected by HSV-1 DNA replication. H3.1 mobilization also requires no HSV-1 proteins. However, H3.1 mobilization peaks early in infection and decreases when HSV-1 DNA replicates. The late decrease does not occur when HSV-1 DNA replication is inhibited. These dynamics are consistent with the assembly of previously mobilized H3.1 into HSV-1 chromatin during viral DNA replication. The dynamics of H3.3 mobilization, in contrast, are consistent with the concomitant mobilization and assembly of H3.3 into HSV-1 nucleosomes throughout infection. The mobilizations of H3.1 and H3.3 are therefore consistent with their differential associations with HSV-1 genomes and provide the first evidence for a role of histone mobilization in the assembly of HSV-1 chromatin. We also show that U2OS cells are defective in mobilizing histone H3.1 in response to infection. These cells allow for the replication of HSV-1 mutants in VP16 and ICP0, indicating that histone mobilization is an important antiviral response that is overcome in part by the activities of viral proteins such as VP16 and ICP0.

## Results

### Histone variants H3.1 and H3.3 are mobilized during HSV-1 infection

**Figure 1:**
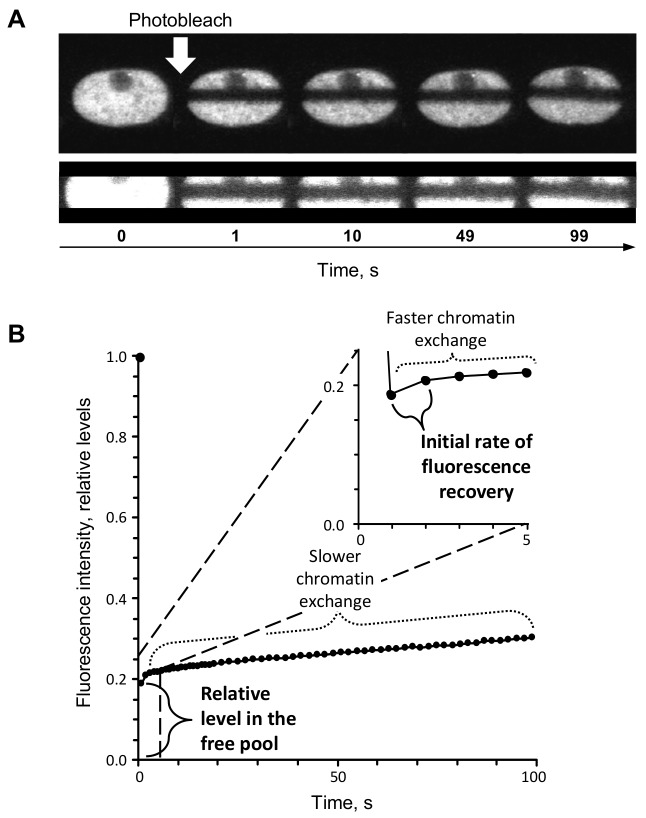
FRAP of GFP-H3 fusion proteins. (**A**) Digital fluorescent micrographs of the nucleus of a cell expressing GFP-H3.3 before and after photobleaching. Vero cells were transfected with plasmids expressing GFP-H3.3. A region passing across the long nuclear axis was photobleached, and the fluorescence recovery in the photobleached region was evaluated. The photobleached region was selected independently of the presence or absence of replication compartments, and thus includes nuclear domains containing cellular and viral DNA. Fluorescence in the photobleached region recovers as the photobleached GFP-histones within this region exchange with the non-photobleached GFP-histones from outside of this region. FRAP was evaluated for only 100 s; therefore, potential contributions from newly synthesized GFP-histones to fluorescence recovery are negligible. The enlargements in the lower panel highlight the photobleached region. (**B**) Line graph of a representative GFP-H3.3 FRAP. The fluorescence intensity of the photobleached region at a given time is normalized to the fluorescence of the entire nucleus at that same time, expressed as a ratio of the normalized fluorescence prior to photobleaching, and plotted against time. The fluorescence intensity is therefore independent of GFP-H3 expression levels. The first data point after photobleaching is a surrogate measure for the levels of free GFP-H3. The subsequent fluorescence recovery is biphasic. The initial faster phase represents those histones that are weakly bound in chromatin and undergoing faster chromatin exchange. As a surrogate measure for this population, we calculated the initial rate of normalized fluorescence recovery (the slope between the normalized fluorescence at the first and second data points after photobleaching; shown in the inset). The second slower phase of fluorescence recovery represents those histones that are more stably bound in chromatin and undergoing slower chromatin exchange.

**Figure 2:**
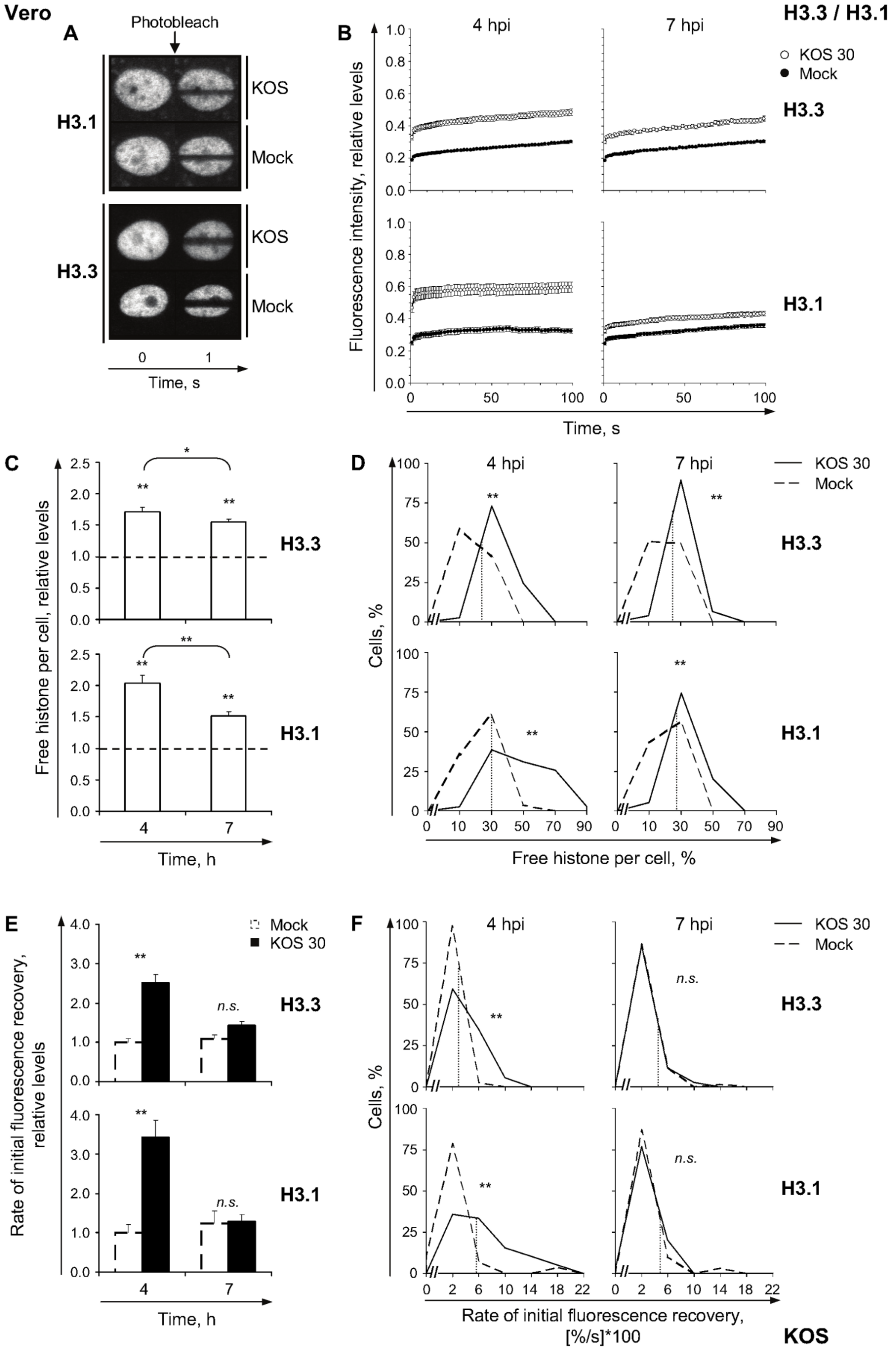
GFP-H3.3 and -H3.1 are mobilized during HSV-1 infection. (**A**) Digital fluorescent micrographs of Vero cells expressing GFP-H3.1 (**H3.1**) or -H3.3 (**H3.3**) at 4 h after infection with 30 PFU/cell of strain KOS (**KOS**) or mock-infection (**Mock**). Images were taken before (0) or 1 s after photobleaching. (**B**) Mobilization of GFP-H3.3 or -H3.1 was examined from 4 to 5 (**4 hpi**) or 7 to 8 (**7 hpi**) hpi by FRAP; error bars, standard errors of the means (SEM). (**C**) Normalized levels of free GFP-H3.3 or -H3.1 relative to mock-infected cells at 4 or 7 hpi, respectively; error bars, SEM; dashed line, normalized levels in mock-infected cells. (**D**) Frequency distribution plots of the percentage of free GFP-H3.3 or -H3.1 in individual cells at 4 or 7 hpi; dotted line, one standard deviation (SD) above the average level of free GFP-H3.3 or -H3.1 in mock-infected cells. (**E**) Average initial rates of normalized fluorescence recovery relative to mock-infected cells at 4 hpi; error bars, SEM. (**F**) Frequency distribution plots of the initial rate of normalized fluorescence recovery of GFP-H3.3 or -H3.1 per individual cell; dotted line, one SD above the average initial rate of GFP-H3.3 or -H3.1 normalized fluorescence recovery in mock-infected cells. **, P<0.01; *, P<0.05; *n.s.*, not significant.

To test whether H3.3 or H3.1 were mobilized during HSV-1 infection, we evaluated the kinetics of fluorescence recovery after photobleaching (FRAP) of GFP-H3.3 or -H3.1 fusion proteins (Figure 1). FRAP is the only technique that directly measures histone mobilization (the mobilized histones are lost during nuclei preparations). First, we evaluated the localization of the GFP-tagged histones. Both GFP-H3 fusion proteins were incorporated into cellular chromatin like endogenous histones. Neither extranuclear fluorescence during interphase nor extrachromosomal fluorescence during mitosis were observed in cells expressing either GFP-H3 fusion protein (Figure S1 A and pre-bleached cells in Figures 1 A and 2 A). Moreover, the levels of GFP-H3.3 or -H3.1 expression did not correlate with their levels in the free pools (i.e. not bound in chromatin), as evaluated by the relative nuclear fluorescence intensity (Figure S1 B; correlation coefficient r^2^ = 0.002 or 0.012, for GFP-H3.3 or -H3.1, respectively).

We also tested whether expression of GFP-H3.3 or -H3.1 affected the progression of HSV-1 infection, by evaluating ICP4 expression and accumulation into replication compartments. ICP4 expression, or accumulation into replication compartments, was not inhibited by GFP-H3.3 or -H3.1 (Figure S2 A, KOS). GFP-H3.3 or -H3.1 were sometimes enriched in and sometimes depleted from replication compartments, as previously shown for other core and linker histones (references [24], [25], [40] and data not shown).

**Figure 6:**
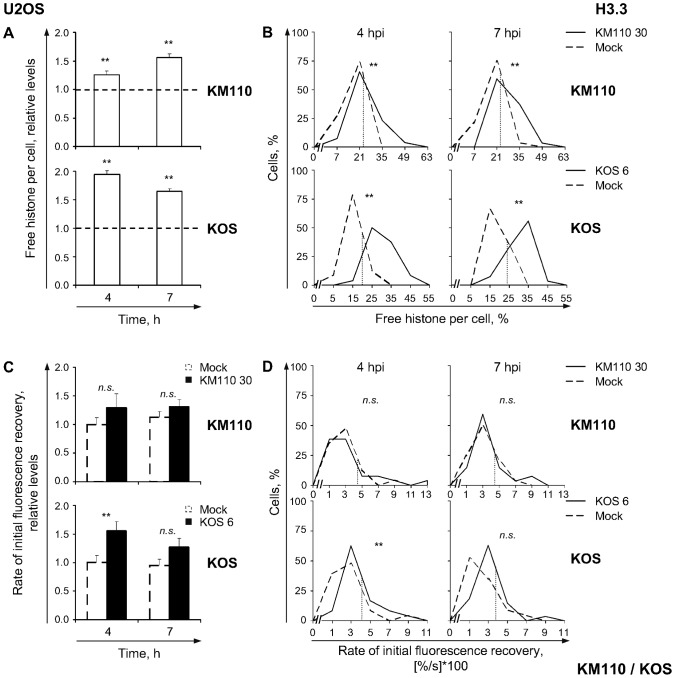
VP16 or ICP0 modulate H3.3 mobilization. (**A**) Average normalized levels of free GFP-H3.3 relative to mock-infected cells at 4 or 7 hpi, respectively. U2OS cells were transfected with plasmids expressing GFP-H3.3. Transfected cells were mock-infected or infected with 30 PFU/cell of strain KM110 (**KM110**) or 6 PFU/cell of strain KOS (**KOS**). Mobilization of GFP-H3.3 was examined from 4 to 5 (**4**) or 7 to 8 (**7**) hpi by FRAP; error bars, SEM; dashed line, average normalized levels of free GFP-H3.3 in mock-infected cells. (**B**) Frequency distribution plots of the percentage of free GFP-H3.3 per individual cell at 4 or 7 hpi; dotted line, one SD above the average level of free GFP-H3.3 in mock-infected cells. (**C**) Average initial rates of normalized fluorescence recovery relative to mock-infected cells at 4 hpi; error bars, SEM. (**D**) Frequency distribution plots of the initial rate of normalized fluorescence recovery of GFP-H3.3 per individual cell; dotted line, one SD above the average initial rate of normalized fluorescence recovery in mock-infected cells. **, P<0.01; *n.s.*, not significant.

The differential binding of H3.3 or H3.1 to HSV-1 DNA as evaluated by ChIP correlates with HSV-1 DNA replication [27]. We therefore evaluated the FRAP kinetics of GFP-H3.3 or GFP-H3.1 before (4 hpi) and during (7 hpi) robust HSV-1 DNA replication (see Figure 6 in reference [41]). The relative normalized fluorescence in the photobleached nuclear region recovered faster in infected cells than in mock-infected cells at 4 or 7 h after infection with 30 PFU/cell of strain KOS (Figure 2). The fluorescence of the photobleached region is normalized to the total nuclear fluorescence at each time, such that recovery is independent of total fluorescence levels. Normalized fluorescence is then expressed relative to the normalized fluorescence before photobleaching (Figure 1 B), again ensuring independence of total fluorescence levels. The relative fluorescence intensity of the photobleached region was greater in HSV-1 infected than in mock-infected cells at all times (Figure 2 A and B). Concomitantly, the fluorescence of the non-bleached region was less intense in HSV-1 infected than in mock-infected cells (Figure 2 A) at all times. The decrease in fluorescence intensity of the non-bleached region reflects the movement of GFP-H3 that is outside of the photobleached region prior to photobleaching moving into the photobleached region during photobleaching (Figure 2 A). This subpopulation of the nuclear histone pool is predominantly composed of freely diffusing molecules. In summary, H3.3 and H3.1 were mobilized during infection.

The FRAP kinetics represent the chromatin exchange of all histones, including those that are, at any given time, not bound in chromatin and therefore available in the free pool, those that are weakly associated with chromatin and therefore undergoing fast chromatin exchange, and those that are stably associated with chromatin and therefore undergoing slow chromatin exchange (Figure 1 B) [42]. Most core histones in non-infected cells are stably bound in chromatin and therefore undergo slow chromatin exchange (Figure 1 B) [42]. This population is not likely available to bind to HSV-1 genomes in timescales relevant to infection. We focused on the histone populations that are most likely available, those in the free pools or undergoing fast chromatin exchange (Figure 1 B).

The fluorescence in the photobleached region was normalized to the total nuclear fluorescence, such that the relative levels are independent of any differences in total fluorescence levels. The normalized levels are then expressed as a fraction of the normalized fluorescence of the same nuclear region prior to photobleaching (Figure 1 B, relative level in the free pool), again ensuring independence of total fluorescence levels.

As a surrogate measure for the level of histones in the free pools, we assessed the normalized relative level of fluorescence in the photobleached region immediately after photobleaching (Figure 1 B). Only freely diffusing histones equilibrate fast enough to enter the photobleached region in such short times (approximately 1s) [42]. As a surrogate measure for the weakly bound histone population, we calculated the initial rate of fast chromatin exchange, the slope between the normalized fluorescence in the photobleached region at the first and second data points after photobleaching (Figure 1 B, initial rate of fluorescence recovery). To account for any potential differences in photobleaching efficiency between experiments, the level of free histones or rates of fast fluorescence recovery in the HSV-1 infected cells was subsequently normalized to those of the mock-infected cells of the same experiment.

The mobilization of H3.3 or H3.1 during HSV-1 infection increased their levels within the free pools. The average relative levels of free H3.3 or H3.1 increased to 171%±7% or 204%±12% at 4 hpi, respectively, and remained increased to 155%±5% or 151%±7% at 7 hpi, respectively (Figure 2 C; *P*<0.01, one-tailed Student's *t* Test). The averages may reflect general increases throughout the cell population, or large increases in only sub-populations of cells. We thus re-evaluated the data by frequency distribution analyses, assessing the levels of free histone per individual cell. The pools of free H3.3 or H3.1 were increased throughout the population of infected cells, as shown by rightward shifts of the unimodal frequency distributions (Figure 2 D). At either time, greater than 70% of infected cells had increased their free pools of H3.3 or H3.1 greater than one standard deviation (S.D.) above the average level in mock-infected cells (dashed lines in Figure 2 D). Only approximately 16% of cells in a normal population would be expected to have these levels. Moreover, the levels of free GFP-H3.3 or -H3.1 did not correlate with their expression levels, as evaluated by relative nuclear fluorescence intensity (Figure S3 A). GFP-H3.1 levels were similar in mock or infected cells, moreover, although levels of GFP-H3.3 tended to be higher in HSV-1 infected cells in some (but not all) experiments (Figure S3 B).

**Figure 3:**
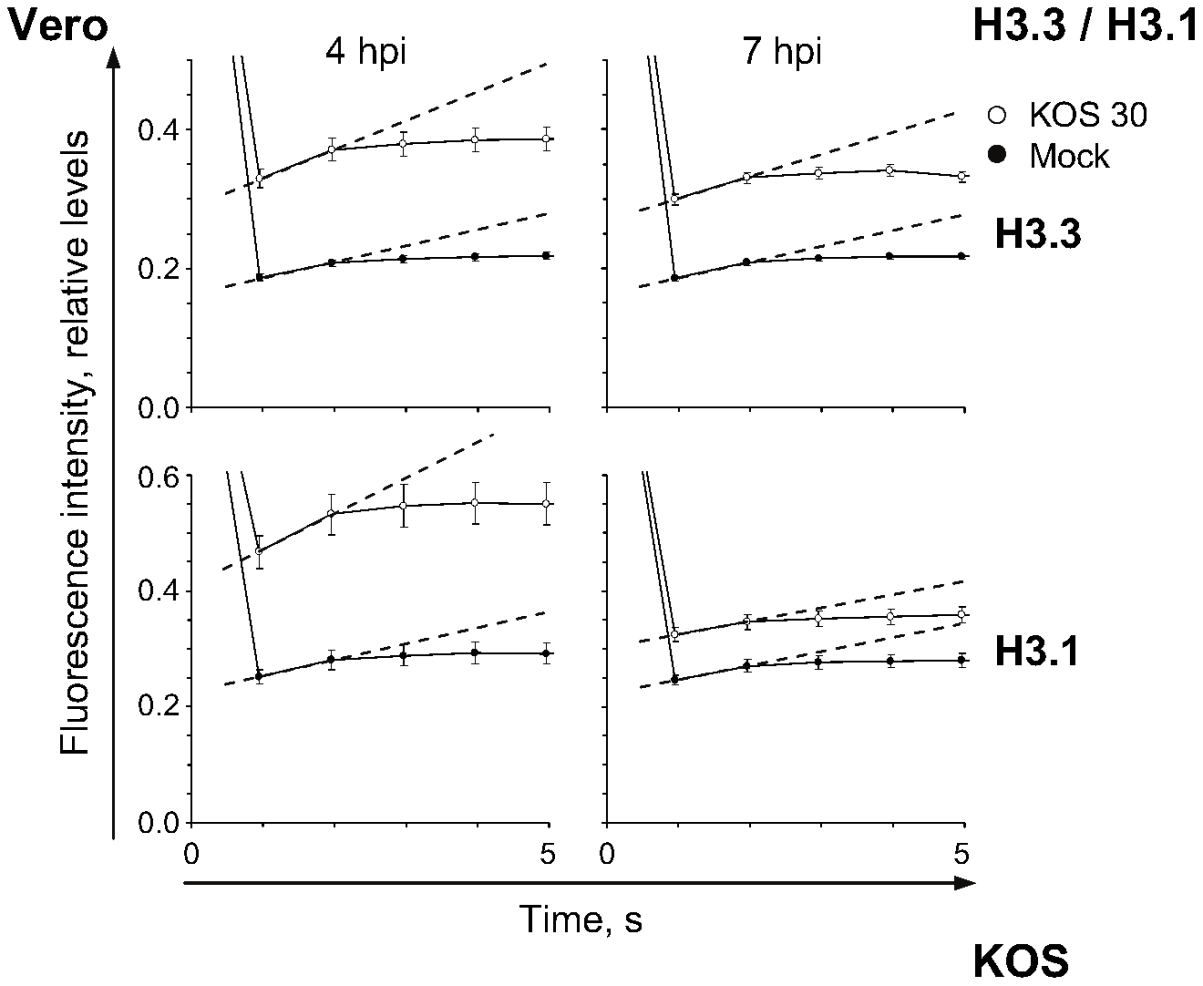
Mobilization increases the fast chromatin exchange rates of GFP-H3.3 or -H3.1. Relative normalized fluorescence intensity of the photobleached region plotted against time; error bars, SEM. Vero cells expressing GFP-H3.3 (**H3.3**) or -H3.1 (**H3.1**) were infected with 30 PFU/cell of strain KOS. Mobilization of GFP-H3.3 or -H3.1 was examined from 4 to 5 (**4 hpi**) or 7 to 8 (**7 hpi**) by FRAP. Graphs re-plotted from Figure 2 to highlight the first 5 s of fluorescence recovery. Dashed lines, slopes between the first and second data points after photobleaching.

Mobilization also increased the average relative rates of H3.3 or H3.1 fast chromatin exchange, to 250%±22% or 342%±43% of mock-infected cells, respectively, at 4 h after infection with 30 PFU/cell of strain KOS (Figure 2 E, Figure 3, 4 hpi; *P*<0.01, one-tailed Student's *t* Test). However, the fast chromatin exchange rates were similar to those in mock-infected cells at 7 h after infection (Figure 2 E, Figure 3, 7 hpi; *P*>0.05, one-tailed Student's *t* test). The rate was increased throughout the cell population at 4 h, shown by rightward shifts of the unimodal frequency distributions (Figure 2 F, 4 hpi). At least 50% of cells had rates of H3.3 or H3.1 fast chromatin exchange greater than one S.D. above the average rate in mock-infected cells (dashed lines in Figure 2 F, 4 hpi). At 7 hpi, the rates in mock or HSV-1 infected cells had similar frequency distributions (Figure 2 F, 7 hpi).

Thus, H3.3 and H3.1 were mobilized during HSV-1 infections, increasing their free pools and increasing their rates of fast chromatin exchange early during infection.

### HSV-1 DNA replication decreases the mobilization of H3.1 but not H3.3

The decrease in H3.1 mobilization from 4 to 7 hpi (*P*<0.01, one-tailed Student's *t* Test) was greater than that of H3.3 (*P*<0.05, one-tailed Student's *t* Test; Figure 2). We next evaluated whether mobilization was associated with HSV-1 DNA replication, using phosphonoacetic acid (PAA) to inhibit the HSV-1 DNA polymerase [43].

**Figure 4:**
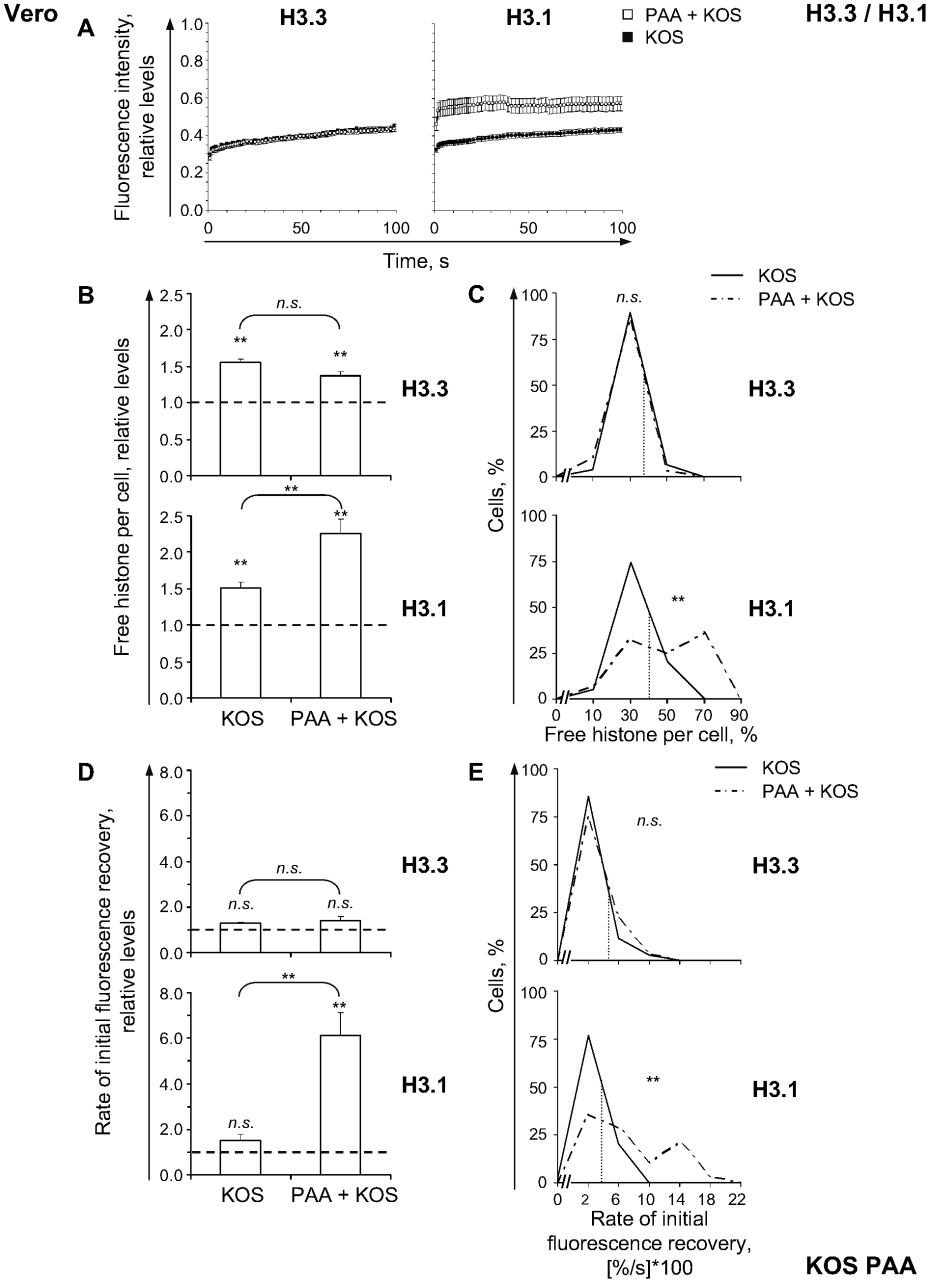
HSV-1 DNA replication decreases mobilization of H3.1 but not of H3.3. (**A**) Relative normalized fluorescence intensity of the photobleached nuclear region plotted against time. Vero cells were transfected with plasmids expressing GFP-H3.3 (**H3.3**) or -H3.1 (**H3.1**). Transfected cells were mock-infected or infected with 30 PFU/cell of strain KOS in the presence of 400 µg/ml of PAA (**PAA+KOS**) or no drug (**KOS**). Mobilization of GFP-H3.3 or -H3.1 was examined from 7 to 8 hpi by FRAP; error bars, SEM. (**B**) Average normalized levels of free GFP-H3.3 or -H3.1 relative to untreated mock-infected cells; error bars, SEM; dashed line, normalized levels in untreated mock-infected cells. (**C**) Frequency distribution plots of the percentage of free GFP-H3.3 or -H3.1 in individual cells; dotted line, one SD above the average level of free GFP-H3.3 or -H3.1 in untreated KOS infected cells. (**D**) Average initial rates of normalized fluorescence recovery relative to untreated mock-infected cells; error bars, SEM; dashed line, average initial rates of normalized fluorescence recovery in untreated mock-infected cells. (**E**) Frequency distribution plots of the initial rate of normalized fluorescence recovery of GFP-H3.3 or -H3.1 in each cell; dotted line, one SD above the average initial rate of normalized fluorescence recovery of GFP-H3.3 or -H3.1 in untreated KOS infected cells. KOS data re-plotted from Figure 2 for comparison. **, P<0.01; *n.s.*, not significant.

H3.3 mobilization did not increase when HSV-1 DNA replication was inhibited (Figure 4 A, H3.3). The normalized relative fluorescence in the photobleached region recovered similarly in cells treated or not with 400 µg/ml of PAA after 7 h of infection with 30 PFU/cell of strain KOS (Figure 4 A, H3.3). Consistently, the increases to the average levels of free H3.3 were similar whether HSV-1 DNA replication was inhibited or not (to 137%±6% or 155%±5%, respectively; Figure 4 B and C, H3.3; *P*>0.05, Tukey's honestly significantly different (HSD) test), and PAA treatment did not drastically affect the increase in free H3.3 throughout the cell population (Figure 4 C, H3.3). The average rates of H3.3 fast chromatin exchange were also similar whether or not HSV-1 DNA replicated (Figure 4 D and E, H3.3; *P*>0.05, Tukey's HSD test). Thus, H3.3 mobilization does not require, nor is it inhibited by, HSV-1 DNA replication (or L proteins).

In contrast, PAA significantly enhanced H3.1 mobilization (Figure 4 A, H3.1). The average free H3.1 increased to 226%±20% in PAA treated cells, but to only 151%±7% in non-treated cells (Figure 4 B, H3.1; *P*<0.01, Tukey's HSD test). Free H3.1 per individual cell displayed a bimodal distribution when HSV-1 DNA replication was inhibited (Figure 4 C, H3.1). One sub-population had similar levels of free H3.1 as non-treated cells, whereas another had even higher levels (Figure 4 C, H3.1), resulting in more than 60% of PAA-treated cells with levels greater than one S.D. above the average level in non-treated infected cells (dashed line in Figure 4 C, H3.1). This increase was not due to higher GFP-H3.1 levels (Figure S3 B, H3.1).

PAA moderately increased the average level of free H3.1 in mock-infected cells (*P*<0.05, one-tailed Student's *t* test; data not shown), but less so than in infected cells (124%±10% versus 226%±20%, respectively; *P*<0.01, Tukey's HSD test).

The average rate of H3.1 fast chromatin exchange was also increased when HSV-1 DNA replication was inhibited, to 613%±97% (Figure 4 D, H3.1; *P*<0.01, one-tailed Student's *t* test), a much greater degree than when HSV-1 DNA replicated (128±18%; Figure 4 D and E, H3.1; *P*<0.01, Tukey's HSD test). The rate per individual cell also displayed a bimodal frequency distribution (Figure 4 E, H3.1). One sub-population of cells had rates of H3.1 fast chromatin exchange similar to those in non-treated cells and the other had even greater rates (Figure 4 E, H3.1; *P*<0.01, Tukey's HSD test), resulting in more than 64% of PAA-treated cells with rates greater than one S.D. above the average in non-treated infected cells (Figure 4 E, H3.1, dashed line; *P*<0.01, Tukey's HSD test).

### H3.3 and H3.1 are less mobilized by replication defective HSV-1 strains

We next evaluated the mobility of H3.3 or H3.1 during infection with a VP16 and ICP0 HSV-1 mutant strain, KM110 [38]. During KM110 infection of Vero cells, IE protein expression is not efficiently initiated (Figure S2 A, KM110) and, consequently, KM110 DNA is not replicated [38].

**Figure 5:**
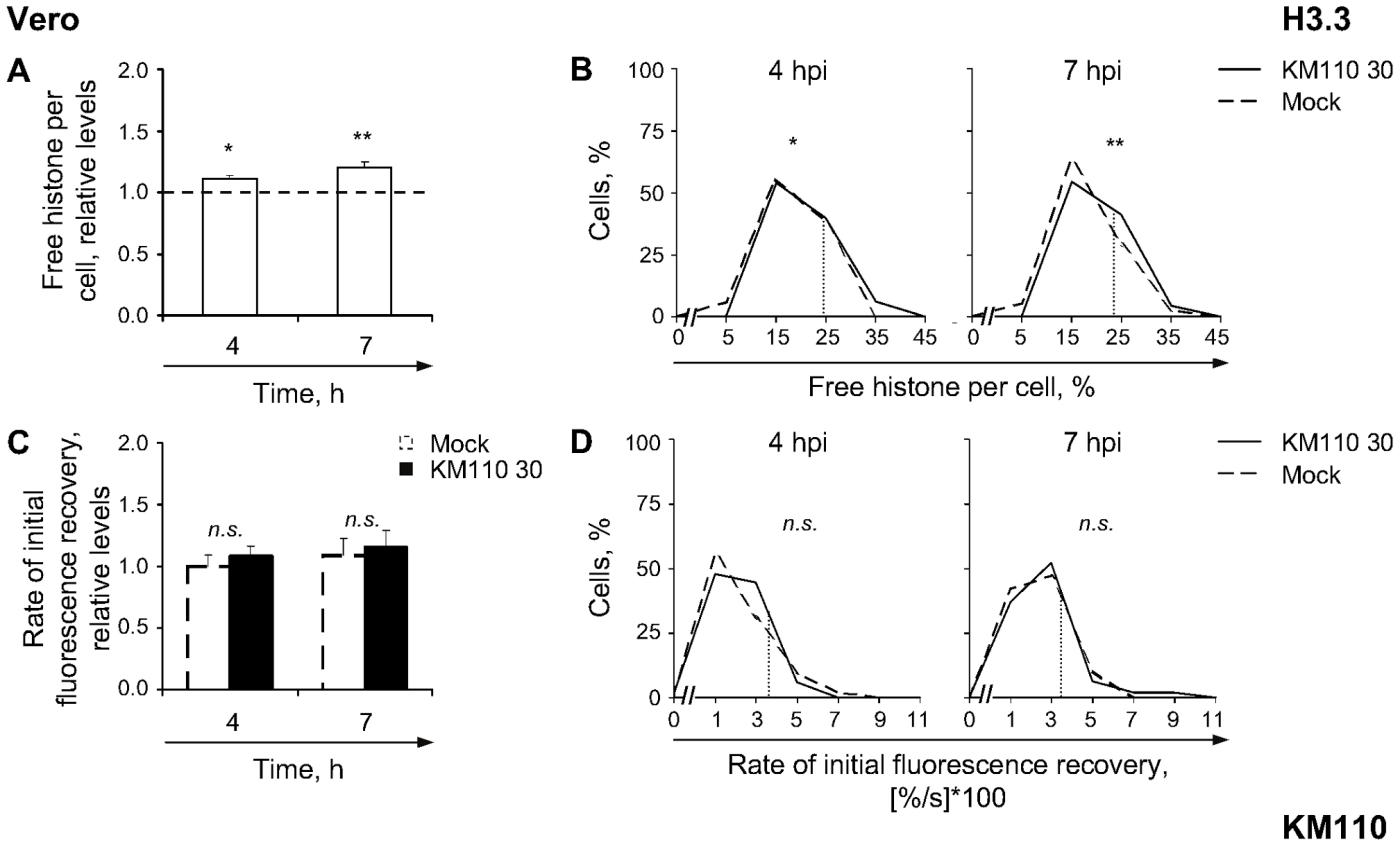
H3.3 is only mobilized to a basal degree during infection with transcription and replication defective strain KM110. (**A**) Average normalized levels of free GFP-H3.3 relative to mock-infected cells at 4 or 7 hpi, respectively. Vero cells were transfected with plasmids expressing GFP-H3.3. Transfected cells were mock-infected or infected with 30 PFU/cell of strain KM110. Mobilization of GFP-H3.3 was examined from 4 to 5 (**4**) or 7 to 8 (**7**) hpi by FRAP; error bars, SEM; dashed line, average normalized level of free GFP-H3.3 in mock-infected cells. (**B**) Frequency distribution plots of the percentage of free GFP-H3.3 per individual cell at 4 or 7 hpi; dotted line, one SD above the average level of free GFP-H3.3 in mock-infected cells. (**C**) Average initial rates of normalized fluorescence recovery relative to mock-infected cells at 4 hpi; error bars, SEM. (**D**) Frequency distribution plots of the initial rate of normalized fluorescence recovery of GFP-H3.3 per individual cell; dotted line, one SD above the average initial rate of normalized fluorescence recovery in mock-infected cells. **, P<0.01; *, P<0.05; *n.s.*, not significant.

H3.3 was still mobilized under these conditions of minimal HSV-1 protein expression and no viral DNA replication, albeit to a limited degree. The average relative level of free H3.3 was increased to only 111%±3% at 4 (*P*<0.05, one-tailed Student's *t* Test), and it remained increased to 121%±5% at 7 (Figure 5 A; *P*<0.01, one-tailed Student's *t* Test), hours after infection with 30 PFU/cell of strain KM110. More than 25% of cells had pools of free H3.3 larger than one S.D. above the average level in mock-infected cells at either time, slightly above the expected 16% (Figure 5 B). The average rate of H3.3 fast chromatin exchange did not increase during KM110 infection (Figure 5 C and D), similarly to when HSV-1 DNA replication was inhibited with PAA. Mobilization of H3.3 without affecting its fast chromatin exchange therefore does not require, nor is it inhibited by, HSV-1 DNA replication (or L proteins).

The average relative increase in free H3.3 was larger in KOS than in KM110 infected cells at 4 or 7 hpi (compare Figures 2 C, H3.3 and 5 A; *P*<0.01, Tukey's HSD). HSV-1 transcription or protein expression, VP16, or ICP0 (or cellular responses to them), therefore enhance H3.3 mobilization.

VP16 and ICP0 interact with many chromatin-modifying proteins [28]–[35], which modulate histone dynamics [4], [44]. U2OS cells complement the transcription and replication defects of HSV-1 mutants in VP16 and ICP0 (Figure S2 B, H3.3 KM110) [39]. However, they do not directly complement any of the known biochemical activities of either protein. In fact, the mechanisms whereby U2OS cells complement such mutants remain largely unknown. The activities of VP16 and ICP0, which indirectly modulate the chromatinization of the viral genomes, would be expected to be less important in cells in which histones were not so readily available to chromatinize infecting viral genomes. We therefore tested whether U2OS cells were defective in mobilizing histone H3.3 or H3.1 (as discussed later).

H3.3 was mobilized in U2OS cells at 4 and 7 h after infection with 30 PFU/cell of strain KM110 (Figure 6, KM110). Mobilization increased the average relative levels of free H3.3, to 126%±6% or 156%±7% at 4 or 7 hpi, respectively (Figure 6 A, KM110; *P*<0.01, one-tailed Student's *t* test). However, the average levels of free H3.3 were lower than in KOS infected U2OS cells (even though KOS infections had to be performed at lower multiplicities due to the obvious nuclear morphologic distortion at higher multiplicities). Free H3.3 was increased to 194%±7% at 4 h after infection with 6 PFU/cell of strain KOS, and remained increased to 164%±5% at 7 h (Figure 6 A, KOS; *P*<0.01, one-tailed Student's *t* test). The increases in free H3.3 in KM110 or KOS infected cells occurred throughout the population, with 70 or 81% of the cells, respectively, having large increases in their free pool of H3.3 at 7 h (Figure 6 B).

The apparent defect in H3.3 mobilization during KM110 infections may reflect its delayed replication. Only approximately 50% of U2OS cells infected with KM110 had ICP4 accumulated into replication compartments at 7 hpi, in comparison to approximately 80% of the KOS infected cells at 4 hpi (Figure S2 B, H3.3 KM110 and KOS). Consistently, the average relative levels of free H3.3 at 7 h after KM110 infection were less than those at 4 h (Figure 6 A; *P*<0.01, Tukey's HSD), but similar to those at 7 h (Figure 6 A; *P*>0.05, Tukey's HSD), after KOS infection. The defect in the early H3.3 mobilization is consistent with VP16 or ICP0 enhancing it.

The average rate of H3.3 fast chromatin exchange was not altered during KM110 infection, or at 7 h after KOS infection (Figure 6 C; *P*>0.05, one-tailed Student's *t* test). It was increased, to 155%±16%, at 4 h after KOS infection (Figure 6 C, KOS; *P*<0.01, one-tailed Student's *t* test). These results are also consistent with VP16 or ICP0 increasing the rate of H3.3 fast chromatin exchange.

VP16 or ICP0 thus enhance the mobilization of H3.3 although they are not required for it. The VP16 and ICP0 within infecting virions are not sufficient to fully mobilize H3.3, however, in that infection with UV-inactivated KOS only marginally mobilized it (it increased the free pool to 121%±5%, but only at 7 h and only in Vero cells, without changes in the cell population distribution or in the rates of histone exchange at any time - data not shown).

**Figure 7:**
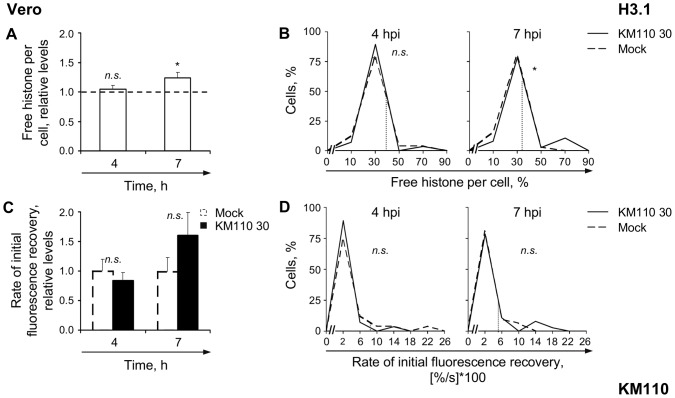
KM110 infection only marginally mobilizes H3.1. (**A**) Average normalized levels of free GFP-H3.1 relative to mock-infected cells at 4 or 7 hpi, respectively. Vero cells were transfected with plasmids expressing GFP-H3.1. Transfected cells were mock-infected or infected with 30 PFU/cell of strain KM110. Mobilization of GFP-H3.1 was examined from 4 to 5 (**4**) or 7 to 8 (**7**) hpi by FRAP; error bars, SEM; dashed line, average normalized levels of free H3.1 in mock-infected cells. (**B**) Frequency distribution plots of the percentage of free GFP-H3.1 per individual cell at 4 or 7 hpi; dotted line, one SD above the average level of free GFP-H3.1 in mock-infected cells. (**C**) Average initial rates of normalized fluorescence recovery relative to mock-infected cells at 4 hpi; error bars, SEM. (**D**) Frequency distribution plots of the initial rate of normalized fluorescence recovery of GFP-H3.1 per individual cell; dotted line, one SD above the average initial rate of normalized fluorescence recovery in mock-infected cells. *, P<0.05; *n.s.*, not significant.

KM110 infection was largely unable to mobilize H3.1 in Vero cells (Figure 7). The average relative level of free, or average rate of fast chromatin exchange of, H3.1 were similar to those in mock-infected cells at 4 h after infection with 30 PFU/cell of strain KM110 (Figure 7 A and C, 4 h; *P*>0.05, one-tailed Student's *t* test). The average relative level of free H3.1 increased to 124%±9%, but only at 7 h after infection (Figure 7 A, 7 h; *P*<0.05, one-tailed Student's *t* test) while the average rate of fast chromatin exchange tended to increase, but didn't reach statistical significance (Figure 7 C, 7 h; *P*>0.05, one-tailed Student's *t* test). The level of free H3.1 and its fast chromatin exchange rate per individual cell had bimodal frequency distributions (Figure 7 B and D), with only the far smallest sub-populations having mobilized it (small solid peaks in Figure 7 B and D). Infection with transcription and replication defective HSV-1 did not mobilize H3.1 nearly as much as when just HSV-1 DNA replication was inhibited. HSV-1 transcription, VP16 or ICP0, expression of other IE or E proteins, or initiation of HSV-1 DNA replication (or cellular responses to them), all of which occur in PAA treated cells but not in KM110 infected ones therefore enhance H3.1 mobilization.

**Figure 8:**
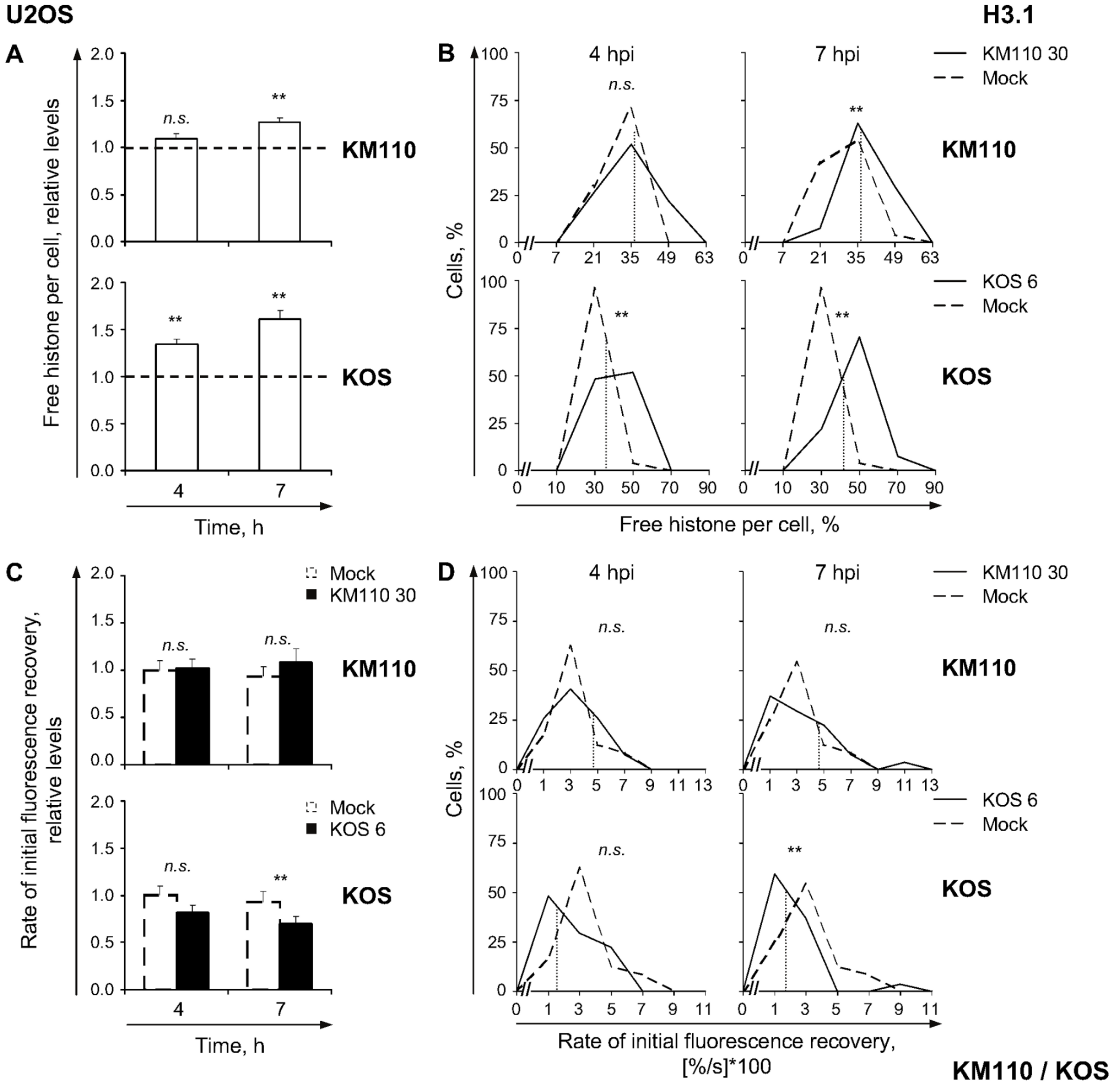
H3.1 is minimally mobilized in U2OS cells. (**A**) Average normalized levels of free GFP-H3.1 relative to mock-infected cells at 4 or 7 hpi, respectively. U2OS cells were transfected with plasmids expressing GFP-H3.1. Transfected cells were mock-infected or infected with 30 PFU/cell of strain KM110 (**KM110**) or 6 PFU/cell of strain KOS (**KOS**). Mobilization of GFP-H3.1 was examined from 4 to 5 (**4**) or 7 to 8 (**7**) hpi by FRAP; error bars, SEM; dashed line, average normalized levels of free GFP-H3.1 in mock-infected cells. (**B**) Frequency distribution plots of the percentage of free GFP-H3.1 per individual cell at 4 or 7 hpi; dotted line, one SD above the average level of free GFP-H3.1 in mock-infected cells. (**C**) Average initial rates of normalized fluorescence recovery relative to mock-infected cells at 4 hpi; error bars, SEM. (**D**) Frequency distribution plots of the initial rate of normalized fluorescence recovery of GFP-H3.1 per individual cell; dotted line, one SD above (KM110) or below (KOS) the average initial rate of normalized fluorescence recovery in mock-infected cells. **, P<0.01; *n.s.*, not significant.

We next evaluated whether U2OS cells mobilized H3.1 in response to infection. The average relative level of free H3.1 at 4 h after infection of U2OS cells with 30 PFU/cell of strain KM110 was similar to that in mock-infected cells (Figure 8 A, KM110; *P*>0.05, one-tailed Student's *t* test). Nevertheless, there was an increased proportion of cells with higher levels of free H3.1 (Figure 8 B, KM110 4 hpi), with almost half (41%) of the cells with levels of free H3.1 greater than one S.D. above the average level in mock-infected cells (almost three times the expected percentage if H3.1 was not mobilized). The average relative level of free H3.1 was increased at 7 h after infection, to 126%±5%, with 52% of cells having large increases in their individual pools of free H3.1 (Figure 8 A and B, KM110 7 h; *P*<0.01, one-tailed Student's *t* test). However, H3.1 levels in the free pool were lower than during KOS infection. The average relative level of free H3.1 increased to 135%±5% at 4 h and further to 146%±5% at 7 h after infection with 6 PFU/cell of strain KOS (Figure 8 A, KOS; *P*<0.01, one-tailed Student's *t* test). Thus, VP16 or ICP0 increase the pool of free H3.1.

The rate of H3.1 fast chromatin exchange was not altered during KM110 infection (Figure 8 C and D, KM110; *P*>0.05, one-tailed Student's *t* test), whereas it tended to decrease at 4 h after KOS infection (to 81%±8%, Figure 8 C, KOS). Although statistical significance was not achieved, 30% of cells had a rate of H3.1 fast chromatin exchange lower than one S.D. below that in mock-infected cells (almost twice as much as expected in a normal distribution; Figure 8 D, KOS). The average rate of H3.1 fast chromatin exchange decreased to 69%±5% at 7 h after infection (Figure 8 C and D; *P*<0.05, one-tailed Student's *t* test). VP16 or ICP0 therefore most likely increase levels of free H3.1 by decreasing its rate of fast chromatin exchange.

### HSV-1 DNA replication does not decrease H3.1 mobilization in U2OS cells

**Figure 9:**
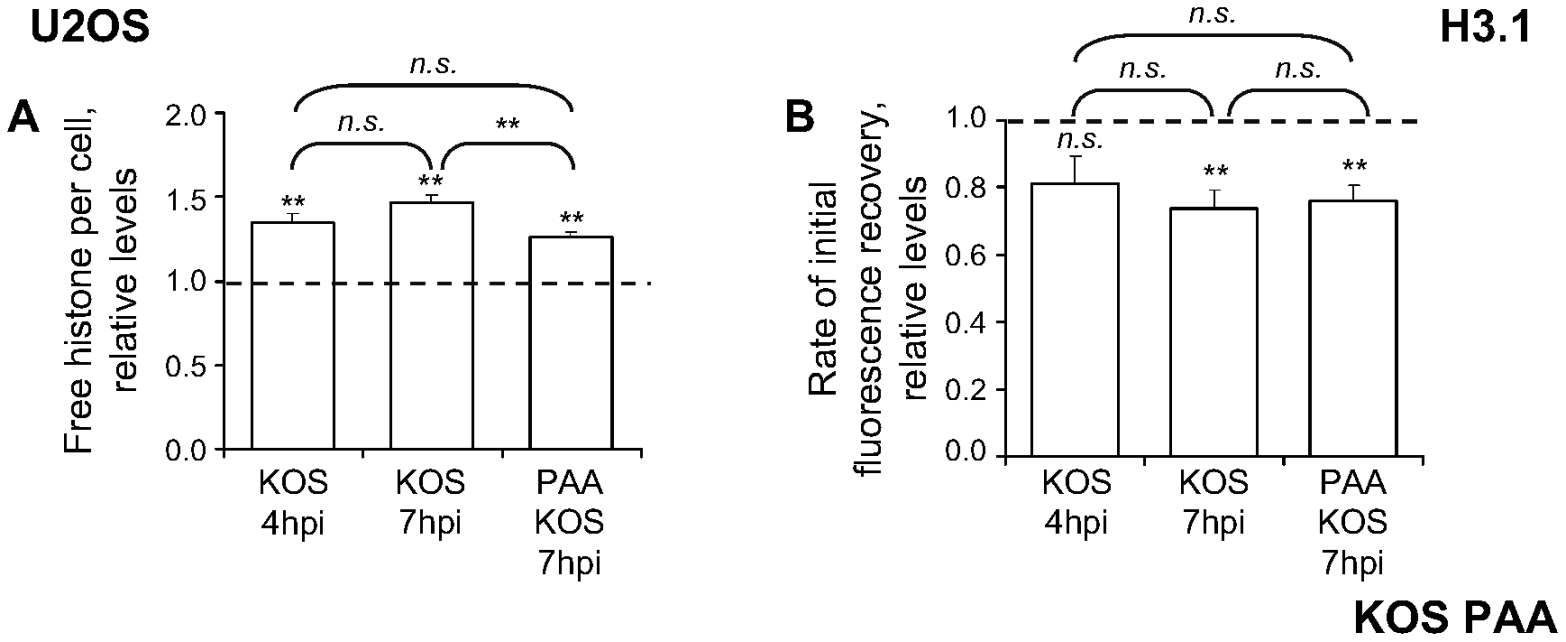
HSV-1 DNA replication does not decrease H3.1 mobilization in U2OS cells. (**A**) Average normalized levels of free GFP-H3.1 relative to untreated mock-infected cells. U2OS cells were transfected with plasmids expressing GFP-H3.1. Transfected cells were mock-infected or infected with 6 PFU/cell of strain KOS in the presence of 400 µg/ml PAA (**PAA KOS**) or no drug (**KOS**). Mobilization of GFP-H3.1 was examined from 4 to 5 (**4 hpi**) or 7 to 8 (**7 hpi**) hpi by FRAP; error bars, SEM; dashed line, average normalized level of free GFP-H3.1 in mock-infected cells. (**B**) Average initial rates of normalized fluorescence recovery of GFP-H3.1 relative to untreated mock-infected cells; error bars, SEM; dashed line, the average initial rate of normalized fluorescence recovery in mock-infected cells. KOS data re-plotted from Figure 8 for comparison. **, P<0.01; *n.s.*, not significant.

H3.1 was differentially mobilized during KOS infection of U2OS or Vero cells (compare Figure 2 C and E, H3.1 to Figure 8 A and C, KOS). We next tested whether inhibition of HSV-1 DNA replication also increased H3.1 mobilization in U2OS cells. H3.1 was mobilized in U2OS cells infected with 6 PFU/cell of strain KOS and treated with 400 µg/ml of PAA (Figure 9), but the average relative level of free H3.1 increased to only 126%±3% (compared to 146%±5% in untreated cells; Figure 9 A; *P*<0.01, one-tailed Student's *t* test). The average level of free H3.1 at 7 h in the presence of PAA was similar to that at 4 h in the absence of PAA (126%±3% vs. 135%±5%, respectively; Figure 9 A; *P*>0.05, Tukey's HSD). The average rates of H3.1 fast chromatin exchange at 7 hpi decreased, to 76%±5% or 69%±5%, respectively, whether or not HSV-1 DNA replication was inhibited (Figure 9 B; *P*<0.01, one-tailed Student's *t* test).

Replication of HSV-1 DNA did not significantly reduce H3.1 mobilization in U2OS cells, indicating that H3.1 is differentially (im)mobilized in Vero and U2OS cells.

### ICP0 enhances H3.1 mobilization but it is not required for it

Pharmacological inhibition of HSV-1 DNA replication enhanced the mobilization of H3.1 in Vero cells (Figure 4, H3.1), whereas infection with transcription and replication defective HSV-1 (KM110) only mobilized it to a limited extent (Figure 7). To test the requirement for HSV-1 transcription, or expression of IE or E proteins (or cellular responses to them), we evaluated H3.1 mobilization in Vero cells infected with the ICP0 mutant strain n212. This strain has the same ICP0 mutation as KM110, but wild-type VP16 [45]. n212 proteins are expressed and its DNA is replicated in Vero cells, although with delayed kinetics. Similar populations of cells infected with 30 PFU/cell of strains KOS or n212 had detectable ICP4 expression (Figure S2 A, KOS versus n212). As expected, however, n212 had delayed replication kinetics (Figure S2 A, KOS versus n212).

**Figure 10:**
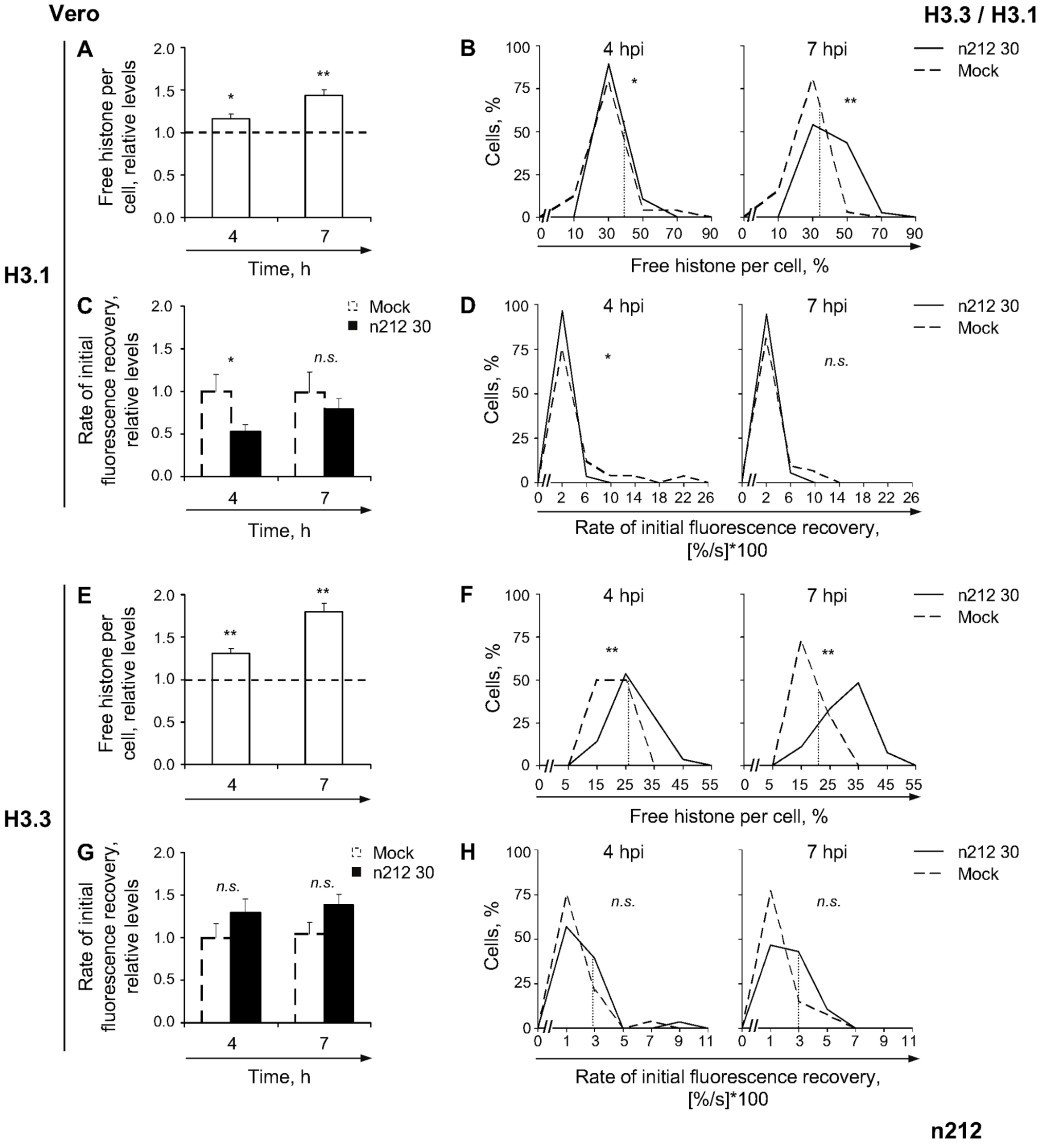
The degree of H3.1 or H3.3 mobilization correlates with the progression of infection. (**A**) Average normalized levels of free GFP-H3.1 relative to mock-infected cells at 4 or 7 hpi, respectively. Vero cells were transfected with plasmids expressing GFP-H3.1 (**H3.1**) or -H3.3 (**H3.3**). Transfected cells were mock-infected or infected with 30 PFU/cell of strain n212. Mobilization of GFP-H3.1 or -H3.3 was examined from 4 to 5 (**4**) or 7 to 8 (**7**) hpi by FRAP; error bars, SEM; dashed line, normalized average level of free GFP-H3.1 in mock-infected cells. (**B**) Frequency distribution plots of the percentage of free GFP-H3.1 per individual cell at 4 or 7 hpi; dotted line, one SD above the average level of free GFP-H3.1 in mock-infected cells. (**C**) Average initial rate of normalized fluorescence recovery relative to mock-infected cells at 4 hpi; error bars, SEM. (**D**) Frequency distribution plots of the initial rate of normalized fluorescence recovery of GFP-H3.1 per individual cell; dashed lines, one SD below the average initial rate of normalized fluorescence recovery in mock-infected cells (not visible because they are at 0 and overlap the y-axis). (**E**) Average normalized levels of free GFP-H3.3 relative to mock-infected cells at 4 or 7 hpi; error bars, SEM; dashed line, normalized average level of free GFP-H3.3 in mock-infected cells. (**F**) Frequency distribution plots of the percentage of free GFP-H3.3 per individual cell at 4 or 7 hpi; dotted line, one SD above the average level of free GFP-H3.1 in mock-infected cells. (**G**) Average initial rate of normalized fluorescence recovery relative to mock-infected cells at 4 hpi; error bars, SEM. (**H**) Frequency distribution plots of the initial rate of normalized fluorescence recovery of GFP-H3.3 per individual cell; dotted line, one SD above the average initial rate of normalized fluorescence recovery in mock-infected cells. **, P<0.01; *, P<0.05; *n.s.*, not significant.

H3.1 was mobilized in Vero cells infected with 30 PFU/cell of strain n212, increasing its average relative free levels to 116%±6% or 144%±6% at 4 or 7 h after infection, respectively (Figure 10 A, H3.1; *P*<0.05, one-tailed Student's *t* test). Sixty-two percent of cells had large increases in free H3.1 at 7 h after infection (Figure 10 B, H3.1). The average rate of fast chromatin exchange decreased to 53%±8% at 4 h (Figure 10 C, H3.1; P<0.05, one-tailed Student's *t* test). The rate still tended to be decreased (to 79%±13%) at 7 h, although statistical significance was not reached (Figure 10 C, H3.1; *P*>0.05, one-tailed Student's *t* test). HSV-1 transcription, IE (other than ICP0) or E proteins, or cellular responses to them, are therefore sufficient to induce H3.1 mobilization.

The average levels of free H3.1 were similarly lower during n212 or KM110 infections (*P*>0.05 at 4 or 7 hpi, Tukey's HSD), which are both defective in ICP0, than during KOS infection. Free H3.1 was less increased even at 7 h after n212 infection than at 4 h after KOS infection. Moreover, the decrease in the average rate of H3.1 fast chromatin exchange at 4 h after n212 infection contrasts with the increase in KOS infection (79%±13% versus 342%±43%, respectively; Figures 2 E, H3.1 4 h, and 10 C, H3.1 4 h). Together, these results indicate that ICP0 enhances H3.1 mobilization by increasing its average rate of fast chromatin exchange.

To further test the role of ICP0 in H3.1 mobilization, we evaluated H3.1 mobility in n212 infected U2OS cells, in which n212 replicates with wild-type kinetics (Figure S2 B, H3.1) [39]. H3.1 was mobilized to at least the same degree during n212 as during KOS infections (Figure S4, H3.1 compare to Figure 8, KOS; *P*>0.05 or *P*<0.01 at 4 or 7 hpi, respectively; Tukey's HSD). ICP0 is therefore less critical to mobilize H3.1 in U2OS than in Vero cells, mirroring its requirements in the HSV-1 replication cycle in each cell type.

### The mobilization of H3.3 is related to HSV-1 transcription and VP16

We next evaluated the effect of ICP0 on H3.3 mobilization. H3.3 was also mobilized in Vero cells infected with 30 PFU/cell of strain n212, increasing the average relative level of free H3.3 to 131%±6% at 4 h after infection, and 180%±10% at 7 h (Figure 10 E; *P*<0.01, one-tailed Student's *t* test). More than 50% of cells had levels of free H3.3 above one S.D. over the average level in mock-infected cells at either time (Figure 10 F). Mobilization also tended to increase the average fast chromatin exchange rate, although it did not reach statistical significance (Figure 10 G; *P*>0.05, one-tailed Student's *t* test). H3.3 was less mobilized at 4 h after n212 than KOS infections (*P*<0.01, Tukey's HSD). However, free H3.3 increased from 4 to 7 h after n212 infection, when its replication delay is compensated (Figure S2 A, H3.3 n212), but not after KOS infections. As a result, the levels of free H3.3 were similar at 7 h after n212 or 4 h after KOS infections (compare Figures 10 E and 2 C, H3.3; *P*>0.05, Tukey's HSD). H3.3 mobilization thus appears to correlate with the progression of infection.

H3.3 is fully mobilized during n212 infection of U2OS cells (Figure S4, H3.3). However, the pool of free H3.3 increased independently of changes to its average rate of fast chromatin exchange (Figure S4, H3.3 compare to Figure 6 C, KOS). Considered together, these results further support the model in which ICP0 modulates H3.3 mobilization by stimulating its fast chromatin exchange (Figure S4, H3.3).

H3.3 was more mobilized during n212 than KM110 infections in Vero (*P*<0.05 or <0.01 at 4 or 7 hpi, respectively, Tukey's HSD) and U2OS cells (*P*<0.01 Tukey's HSD; compare Figure S4 E to Figure 6 A). However, n212 expresses all proteins and replicates in both cell types whereas KM110 does so only in U2OS cells. Therefore, VP16 contributes to H3.3 mobilization in the presence or absence of other viral proteins.

## Discussion

Here, we report that core histones H3.3 and H3.1 are mobilized during HSV-1 infection. The mobilization of H3.3 and H3.1 increases their free pools and alters their rates of fast chromatin exchange. Mobilization of H3.1, but not of H3.3 decreases with HSV-1 DNA replication, which is consistent with the models in which a population of previously mobilized H3.1 is immobilized by assembly into HSV-1 chromatin during HSV-1 DNA replication. Alternatively, H3.1 may be promptly displaced from the infecting viral genomes, but not from the replicating ones, by the HSV-1 transcription activators such as ICP0 and VP16. The differential mobilization of H3.3 and H3.1 is fully consistent with their known temporal associations with HSV-1 genomes [27], and provides the first evidence that histone mobilization relates to viral chromatin assembly. Intriguingly, HSV-1 DNA replication does not decrease H3.1 mobilization in U2OS cells, which are permissive for ICP0 or VP16 mutants. This defect in the immobilization of histone H3.1 in U2OS cells may reflect a defect in assembling silencing chromatin on the viral genomes in these cells. The chromatin-disrupting activities of VP16 and ICP0 would then be less required in these cells, resulting in the observed complementation (as discussed below).

The mobilization of histones provides a process whereby histones assembled in (and exchanging with) cellular chromatin become available for assembly into HSV-1 chromatin. The differential mobilization of H3.3 and H3.1 reported herein, which is consistent with their differential assembly into HSV-1 chromatin [27], provides the first example relating nuclear histone dynamics to the composition of viral chromatin. Moreover, the differential mobilizations of H3.3 and H3.1, together with the results from ChIP assays [27], suggest that there are two mechanisms for HSV-1 chromatin assembly. Mobilization and association of H3.3 with HSV-1 DNA is consistent with its assembly into HSV-1 chromatin mainly via DNA replication-independent mechanisms. Mobilization and association of H3.1 with HSV-1 DNA is consistent with its assembly into HSV-1 chromatin mainly via DNA replication-dependent mechanisms. H3.1 would thus not be expected to significantly associate with HSV-1 genomes in the absence of HSV-1 DNA replication. Appropriately, H3.3 is the H3 variant expressed in terminally differentiated neurons [46], and therefore available to chromatinize non-replicated HSV-1 genomes during the establishment of latency. Alternatively, the association of H3.1 with the infecting viral genomes may be specifically disrupted by the viral transactivators such as VP16 and ICP0, to prevent silencing.

The pools of free H3.3 and H3.1 were both increased early during infection (to 170% and 200%, respectively; Figure 2 C, 4 hpi). However, HSV-1 DNA is primarily associated with H3.3 at this time [27]. This association would be consistent with the transcription-associated assembly of H3.3 into cellular chromatin. However, H3.3 (and other core histones) associate with HSV-1 genes of all kinetic classes within the first hour of infection [19]. Most HSV-1 genes are therefore initially assembled into chromatin independently of their individual transcription. The early HSV-1 chromatin assembly may rather be analogous to that of the sperm pronucleus at fertilization, when the protamines bound to the sperm DNA are replaced with H3.3-containing nucleosomes by HIRA and chromodomain helicase DNA binding protein 1 (CHD) [47], [48]. Mutations of HIRA or CHD are detrimental to the formation of the male chromatin, and consequently the male DNA remains inaccessible [48], [49]. Likewise, HSV-1 genomes are first assembled into nucleosomes containing H3.3, and knockdown of HIRA decreases the association of H3.3 with the viral genomes and decreases HSV-1 protein expression and DNA replication [27].

The dynamics of viral chromatin are proving important in the regulation of HSV-1 gene expression (reviewed in [50]). Whilst cellular mechanisms promote the establishment of repressive viral chromatin to silence viral gene expression, viral mechanisms counteract silencing and promote the establishment of transcriptionally active viral chromatin. The initial assembly of H3.3, as opposed to H3.1, into HSV-1 nucleosomes may be induced by the virus to facilitate chromatin dynamics to circumvent silencing. The “active” posttranslational modifications of H3.3 [51] may recruit the RNA polymerase transcription complex, while unstable H3.3-containing nucleosomes [8] may facilitate its access to the viral DNA. The viral transactivators such as VP16 and ICP0 may actively displace H3.1 from the viral genomes to allow the assembly of H3.3 containing nucleosomes early in infection. Nonetheless, the subsequent assembly of H3.1 into HSV-1 nucleosomes is also important. Depletion of Asf1b, the H3/H4 chaperone that interacts with CAF-1 during DNA replication-dependent chromatin assembly, reduces the levels of HSV-1 DNA and L proteins [52].

H3.1 is mobilized prior to robust HSV-1 DNA replication (see Figure 6 in reference [41]). However, infection with the transcription and replication defective strain KM110 largely failed to mobilize H3.1, indicating that it is mobilized in response to viral transcription, IE or E proteins, or VP16. Despite its early mobilization, moreover, H3.1 does not significantly associate with HSV-1 genomes in the absence of HSV-1 DNA replication [27]. The mechanisms that mobilize H3.1 away from cellular (or viral) chromatin therefore differ from those that stably assemble it into the viral chromatin concomitantly with HSV-1 DNA replication.

H3.1 was differentially mobilized in the two cell lines evaluated, Vero and U2OS (compare Figure 2, H3.1 and Figure 8, KOS), which are non-permissive or permissive, respectively, for the replication of HSV-1 mutants in two proteins that promote chromatin remodeling, ICP0 and VP16. Mobilization increased the rate of H3.1 fast chromatin exchange in Vero cells (Figure 2, H3.1) but decreased it in U2OS cells (Figure 8, KOS). Moreover, inhibition of HSV-1 DNA replication increased H3.1 mobilization in Vero but not in U2OS cells (compare Figure 4, H3.1 and Figure 9). The mobilization of H3.1 in Vero cells is consistent with promotion of H3.1 release from cellular chromatin to increase its levels in the free pool, for later assembly into HSV-1 chromatin. Mobilization in U2OS cells, however, is more consistent with preventing the already free H3.1 from re-binding to (viral) chromatin. U2OS cells complement the replication defects of HSV-1 mutants in VP16 and ICP0, proteins which induce chromatin modifications. The different mobilization of H3.1 in U2OS and Vero cells provides the first evidence that U2OS cells are defective in a response to infection that is active in Vero cells. Consistently, ICP0 appears to stimulate H3.1 mobilization in Vero but not U2OS cells (compare Figure 10, H3.1 to Figure S5, H3.1), which may well relate to the differences in permissivity of each cell type for the replication of ICP0 mutant strains. The different effects of ICP0 on H3.1 mobilization and viral replication in the two cell lines may also reflect H3.1 being less efficiently mobilized away from the cellular chromatin in U2OS than in Vero cells. The inability of U2OS cells to efficiently mobilize H3.1 would place less reliance on the chromatin remodeling activities of ICP0 in these cells.

HSV-1 nucleosomes contain histones with repressive modifications when the viral genes are not expressed (reviewed in [15]). VP16 directly recruits histone acetyltransferases (KATs) [30], [34], while its associated protein, HCF-1, recruits the histone methyltransferases (HMTs) SET1 and MLL1 and the histone demethylases LSD1 and JMJD [53]–[56]. These enzymes promote the modification of the viral histones with transcription activating marks. Whereas siRNA depletion of KATs does not inhibit IE gene expression, siRNA depletion of LSD1, or LSD inhibitors, does and also increases the inhibitory modifications on the histones associated with IE promoters [31], [53], [55], [57].

The recruitment of chromatin modifying proteins to HSV-1 genomes may also influence histone mobility. In addition to VP16 recruitment of KATs [30], [34], ICP0 disrupts histone deacetylase (HDAC) activity [28], [29], [32]. Together, VP16 and ICP0 could promote the acetylation of a pool of histones, increasing their chromatin exchange. ICP0 and VP16 also reduce stable (total) H3 binding to HSV-1 genomes [30], [37], [58], [59], further supporting a model in which they promote (total) H3 chromatin exchange. ICP0 also stimulates the rate of H3 fast chromatin exchange, possibly by decreasing the propensity of H3 to bind to the viral genomes. However, neither ICP0 nor VP16 is necessary to mobilize (total) H3, indicating that other HSV-1 proteins most likely also induce histone mobilization. We are working to address this model.

The mobilization of histones during HSV-1 infection requires chromatin to be assembled and disassembled, processes which involve histone chaperones. Of the four known H3 chaperones [9], [11], [12], three alter HSV-1 gene expression or replication [27], [52], [60]. This involvement highlights the importance of viral nucleosome turnover in the regulation of HSV-1 transcription and replication.

In summary, the results presented herein strongly support the models in which histones are mobilized away from the cellular genome to form silencing chromatin on the viral genomes, but the viral transcription activators (such as ICP0 and VP16) further mobilize histones away from the viral genomes to prevent silencing. The chromatin dynamics during HSV-1 infection, including the mobilization of histones, are an exciting new area of epigenetic regulation of viral gene expression.

## Materials and Methods

### Cells, viruses, and drugs

African green monkey (Vero) cells were maintained at 37°C in 5% CO_2_ in Dulbecco's Modified Eagle's Medium (DMEM) supplemented with 5% fetal bovine serum (FBS). Osteosarcoma (U2OS) cells, a generous gift from Dr. J. Smiley (University of Alberta) were maintained at 37°C in 5% CO_2_ in DMEM supplemented with 10% FBS. Wild-type HSV-1, strain KOS (passage 10), and mutant strains n212 (the late Dr. P. Schaffer; Harvard Medical School) and KM110 (Dr. J. Smiley; University of Alberta) are described [38], [45], [61]. Viral stocks were prepared and titrated by standard plaque assay as described [24], [25]. Phosphonoacetic acid (PAA; Sigma) was prepared in DMEM as a 100 mg/ml stock at neutral pH, stored in aliquots at −20°C, and used at 400 µg/ml.

### Plasmids

The cDNA sequence encoding H3.1, which is fully conserved in mouse (NP_659531) and human (NP_003522), was obtained from the Riken Mouse cDNA library [62], [63]. H3.1 was PCR amplified with the sense (5′- TGGGAGATCTGAGTGG GTTGCTATGG) and antisense (5′- TTTGGTCGACAGCTGGCACGACAGGT) primers. The amplified sequence was digested with PvuII and BglII for directional in-frame cloning into pEGFP-C1 previously digested with SmaI and BglII.

pEGFP-H3.3 was a generous gift from Dr. John Th'ng (Northern Ontario School of Medicine). Human H3.3, with flanking 5′ BglII and 3′ EcoR1 restriction sites, was sub-cloned into pEGFP-C1.

### Transfection

Vero and U2OS cells were transfected with Lipofectamine 2000 (Invitrogen) as described [24], [25]. After transfection, cells were incubated at 37°C for at least 12 (GFP-H3.3) or 24 (GFP-H3.1) h before any other procedure.

### HSV-1 infection

Transfected cells were seeded onto coverslips for FRAP or immunofluorescence as described [24], and incubated at 37°C in 5% CO_2_ for at least 4 h before infection. Infections were done as described [25]. Inoculum was removed 1 h after addition. Cells were then washed and overlayed with fresh 37°C DMEM supplemented with 5% (Vero) or 10% (U2OS) FBS and incubated in 5% CO_2_ at 37°C until being subjected to FRAP or any additional procedure.

### Fluorescence recovery after photobleaching (FRAP)

Histone mobilization was evaluated from 4 to 5 or 7 to 8 hours post infection (hpi) as described [24], [25]. A region passing approximately across the middle of the nucleus was photobleached. The photobleached region included nuclear domains containing cellular and viral DNA. Sixty fluorescent and DIC images were collected for each cell at timed intervals from before to after bleaching (one image per second for the first 20 s, followed by 20 images at 2 s intervals then 20 images at 1 s interval). The fluorescence of the photobleached region and of the entire nucleus was measured at each time. The fluorescence of the photobleached region was normalized to the total nuclear fluorescence, ensuring independence of total fluorescence levels. It was next expressed as a percentage of the normalized fluorescence of the same region before photobleaching (Figure 1), further ensuring independence of total fluorescence levels. Fluorescence in the photobleached region is recovered as bleached GFP-histones exchange for non-bleached GFP-histones. FRAP was only measured for 100 seconds; potential contributions by newly synthesized GFP-core histones are not relevant.

The normalized fluorescence intensity of the photobleached nuclear region at the first time after photobleaching was used as a surrogate measure for the levels of histones available in the free pools (i.e., not bound in chromatin; Figure 1) [42]. The slope between the normalized fluorescence at the first and second data points after photobleaching, representing the initial rate of fluorescence recovery, was used as a surrogate measure for the rate of fast chromatin exchange (Figure 1).

### Image preparation

Fluorescent images (512 by 512; 12 bit) were analyzed with Zeiss LSM software. Images were cropped and their contrast and brightness were adjusted for figure preparation using GIMP 2.

### Statistics

For comparisons involving only two samples, we used a one-tailed Student's *t* Test. For comparisons involving multiple samples, we used ANOVA to identify if any sample was different from the rest. The results for which ANOVA indicated there were differences were further evaluated by post hoc Tukey's Honestly Significant Difference (HSD) to identify the samples that differed from each other. To evaluate the association between the level of GFP-histone expression and the level in the free pools of individual cells the square of the correlation coefficient (r^2^) was calculated.

## Supporting Information

Figure S1 **GFP-H3.3 or -H3.1 incorporate in chromatin and their expression levels don't correlate with free pools.** (**A**) Digital fluorescent images of cells expressing GFP-H3.3 (**H3.3**) or -H3.1 (**H3.1**) as they go through mitosis. The GFP-H3 fusion proteins are assembled in chromatin as endogenous histones and no extra-chromosomal fluorescence is observed. The H3.1 expressing cells divided along in the Z-axis, hence chromatid separation during anaphase is not visible. Furthermore, only one nucleus is visible in the plane shown in telophase, the second nucleus moves into the visible plane in the subsequent interphase images. (**B**) Dot plots of the level of free GFP-H3 per individual cell plotted against normalized fluorescence intensity. Vero cells were transfected with plasmids encoding GFP-H3.3 (**H3.3**) or -H3.1 (**H3.1**). Transfected cells were mock infected at least 12 (H3.3) or 24 (H3.1) hours after transfection. Free GFP-H3.3 or -H3.1 was evaluated by FRAP 4 to 5 or 7 to 8 hours later. Correlation coefficients, H3.3 r^2^ = 0.002; H3.1 r^2^ = 0.012.
(PDF)

Figure S2 **Expression of GFP-H3.3 or -H3.1 does not inhibit ICP4 expression or accumulation into replication compartments.** Percentage of HSV-1 infected cells transfected with either GFP-H3.3 or -H3.1 and expressing ICP4 as nuclear diffuse or in replication compartments. Vero (**A**) or U2OS (**B**) cells were transfected with plasmids expressing GFP-H3.3 (**H3.3**) or -H3.1 (**H3.1**) fusion proteins. At least 12 (H3.3) or 24 (H3.1) hours after transfection, cells were infected with 6 (U2OS) or 30 (Vero) PFU/cell of strain KOS (**KOS**), or 30 PFU/cell of strain n212 (**n212**), or KM110 (**KM110**). Infected cells were fixed at 4.5 (**4**) or 7.5 (**7**) hpi and stained for ICP4. Nuclear expression of ICP4 and its accumulation in replication compartments in cells in which GFP-H3.3 or -H3.1 were expressed (**+**) or not (**−**) was evaluated by fluorescence microscopy. Small replication compartments occupied, alone or in combination, less than half of the nuclear area. Large replication compartments occupied, alone or in combination, at least half of the nucleus.
(PDF)

Figure S3 **Levels of free GFP-H3.3 or -H3.1 in infected cells do not correlate with expression levels.** (**A**) Levels of free GFP-H3 per individual cell plotted against normalized fluorescence intensity. Vero cells were transfected with plasmids expressing GFP-H3.3 (**H3.3**) or -H3.1 (**H3.1**). At least 12 (H3.3) or 24 (H3.1) hours after transfection, cells were mock-infected or infected with 30 PFU/cell of strain KOS and treated (**+**) or not (**−**) with 400 µg PAA. Levels of free GFP-H3.3 or -H3.1 were evaluated 4 to 5 (**4 hpi**) or 7 to 8 (**7 hpi**) hours later by FRAP. Correlation coefficients, H3.3 r^2^ = 0.104, 0.002, or 0.054 at 4, 7, or 7 hpi with PAA treatment, respectively; H3.1 r^2^ = 0.096, 0.022, or 0.006 at 4, 7, or 7 hpi with PAA treatment, respectively. (**B**) Western blots showing the expression levels of GFP-H3.3 or -H3.1 fusion proteins and endogenous H3. Cells were harvested at 4 hpi, nuclear and cytoplasmic extracts were resolved by SDS-PAGE, and the levels of GFP or H3 expression were analyzed by Western Blot. The average ratio of GFP-H3 to endogenous H3 signal intensities calculated from 3 (H3.3), 4 (H3.1), or 2 (H3.1 with PAA) experiments is presented; dashes, ratio could not be calculated (at least one value is 0).
(PDF)

Figure S4 **The degree of GFP-H3.1 or -H3.3 mobilization in U2OS cells correlates with infection progression.** (**A**) Average normalized levels of free GFP-H3.1 relative to mock-infected cells at 4 or 7 hpi, respectively. U2OS cells were transfected with plasmids expressing GFP-H3.1 (**H3.1**) or -H3.3 (**H3.3**). Transfected cells were mock-infected or infected with 30 PFU/cell of strain n212. Mobilization of GFP-H3.1 or -H3.3 was examined from 4 to 5 (**4**) or 7 to 8 (**7**) hpi by FRAP; error bars, SEM; dashed line, normalized average level of free GFP-H3.1 in mock-infected cells. (**B**) Frequency distribution plots of the percentage of free GFP-H3.1 per individual cell at 4 or 7 hpi; dotted line, one SD above the average level of free GFP-H3.1 in mock-infected cells. (**C**) Average initial rate of normalized fluorescence recovery relative to mock-infected cells at 4 hpi; error bars, SEM. (**D**) Frequency distribution plots of the initial rate of normalized fluorescence recovery of GFP-H3.1 per individual cell; dotted line, one SD below the average initial rate of normalized fluorescence recovery in mock-infected cells. (**E**) Average normalized levels of free GFP-H3.3 relative to mock-infected cells at 4 or 7 hpi, respectively; error bars, SEM; dashed line, normalized average level of free GFP-H3.3 in mock-infected cells. (**F**) Frequency distribution plots of the percentage of free GFP-H3.3 per individual cell at 4 or 7 hpi; dotted line, one SD above the average level of free GFP-H3.1 in mock-infected cells. (**G**) Average initial rate of normalized fluorescence recovery relative to mock-infected cells at 4 hpi; error bars, SEM. (**H**) Frequency distribution plots of the initial rate of normalized fluorescence recovery of GFP-H3.3 per cell; dotted line, one SD above the average initial rate of normalized fluorescence recovery in mock-infected cells. **, P<0.01; *n.s.*, not significant.
(PDF)

Methods S1 **Supplementary materials and methods information.**
(DOCX)
