# Correction:The Differential Mobilization of Histones H3.1 and H3.3 by Herpes Simplex Virus 1 Relates Histone Dynamics to the Assembly of Viral Chromatin

**DOI:** 10.1371/journal.ppat.1005575

**Published:** 2016-04-13

**Authors:** Kristen L. Conn, Michael J. Hendzel, Luis M. Schang

## Notice of Republication

Fig. 1 was published in error. The error was corrected in the HTML and PDF versions of this article on March 23, 2016, and the corrected figure is available in the supporting information of this article.

## Supporting Information

S1 File Corrected Fig. 1 (TIF)

## References

[1] Conn KL, Hendzel MJ, Schang LM (2013) The Differential Mobilization of Histones H3.1 and H3.3 by Herpes Simplex Virus 1 Relates Histone Dynamics to the Assembly of Viral Chromatin. PLoS Pathog 9(10): e1003695 doi:10.1371/journal.ppat.1003695 24130491 10.1371/journal.ppat.1003695 PMC3795045

