# Supplementary material for: The Differential Mobilization of Histones H3.1 and H3.3 by Herpes Simplex Virus 1 Relates Histone Dynamics to the Assembly of Viral Chromatin
Source: PLoS Pathog. 2013 Oct 10;9(10):e1003695. doi: 10.1371/journal.ppat.1003695 (PMC3795045; doi:10.1371/journal.ppat.1003695)
Supplement: Figure S1 — GFP-H3.3 or -H3.1 incorporate in chromatin and their expression levels don't correlate with free pools. (A) Digital fluorescent images of cells expressing GFP-H3.3 (H3.3) or -H3.1 (H3.1) as they go through mitosis. The GFP-H3 fusion proteins are assembled in chromatin as endogenous histones and no extra-chromosomal fluorescence is observed. The H3.1 expressing cells divided along in the Z-axis, hence chromatid separation during anaphase is not visible. Furthermore, only one nucleus is visible in the plane shown in telophase, the second nucleus moves into the visible plane in the subsequent interphase images. (B) Dot plots of the level of free GFP-H3 per individual cell plotted against normalized fluorescence intensity. Vero cells were transfected with plasmids encoding GFP-H3.3 (H3.3) or -H3.1 (H3.1). Transfected cells were mock infected at least 12 (H3.3) or 24 (H3.1) hours after transfection. Free GFP-H3.3 or -H3.1 was evaluated by FRAP 4 to 5 or 7 to 8 hours later. Correlation coefficients, H3.3 r2 = 0.002; H3.1 r2 = 0.012. (PDF) [file ppat.1003695.s001.pdf]

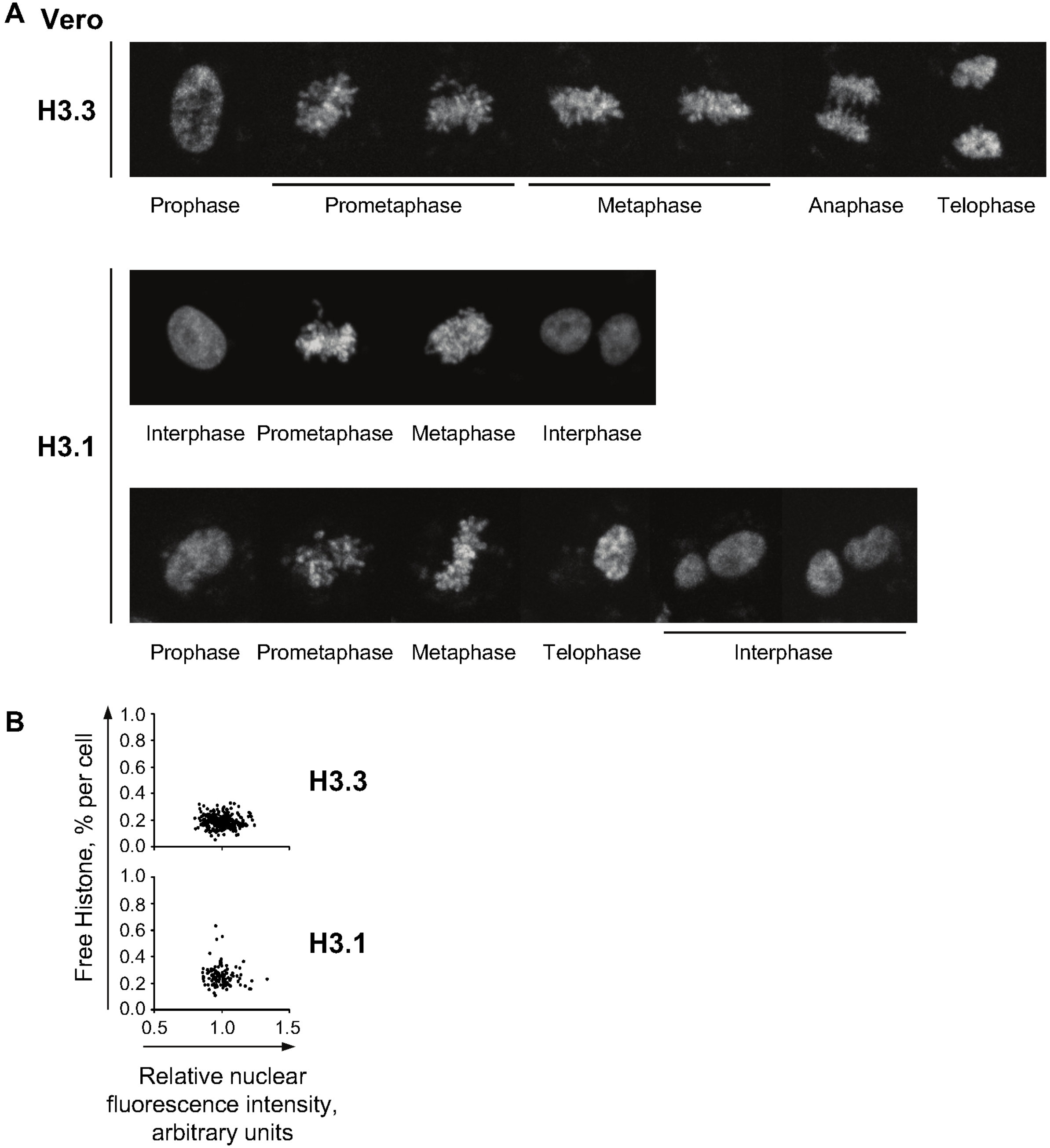
A
Vero
H3.3
Prophase
Prometaphase
Metaphase
Anaphase
Telophase
H3.1
Interphase
Prometaphase
Metaphase
Interphase
Prophase
Prometaphase
Metaphase
Telophase
Interphase
B
Free Histone, % per cell
1.0
0.8
0.6
0.4
0.2
0.0
H3.3
H3.1
0.5
1.0
1.5
Relative nuclear fluorescence intensity, arbitrary units
