# Supplementary material for: The Differential Mobilization of Histones H3.1 and H3.3 by Herpes Simplex Virus 1 Relates Histone Dynamics to the Assembly of Viral Chromatin
Source: PLoS Pathog. 2013 Oct 10;9(10):e1003695. doi: 10.1371/journal.ppat.1003695 (PMC3795045; doi:10.1371/journal.ppat.1003695)
Supplement: Figure S2 — Expression of GFP-H3.3 or -H3.1 does not inhibit ICP4 expression or accumulation into replication compartments. Percentage of HSV-1 infected cells transfected with either GFP-H3.3 or -H3.1 and expressing ICP4 as nuclear diffuse or in replication compartments. Vero (A) or U2OS (B) cells were transfected with plasmids expressing GFP-H3.3 (H3.3) or -H3.1 (H3.1) fusion proteins. At least 12 (H3.3) or 24 (H3.1) hours after transfection, cells were infected with 6 (U2OS) or 30 (Vero) PFU/cell of strain KOS (KOS), or 30 PFU/cell of strain n212 (n212), or KM110 (KM110). Infected cells were fixed at 4.5 (4) or 7.5 (7) hpi and stained for ICP4. Nuclear expression of ICP4 and its accumulation in replication compartments in cells in which GFP-H3.3 or -H3.1 were expressed (+) or not (−) was evaluated by fluorescence microscopy. Small replication compartments occupied, alone or in combination, less than half of the nuclear area. Large replication compartments occupied, alone or in combination, at least half of the nucleus. (PDF) [file ppat.1003695.s002.pdf]

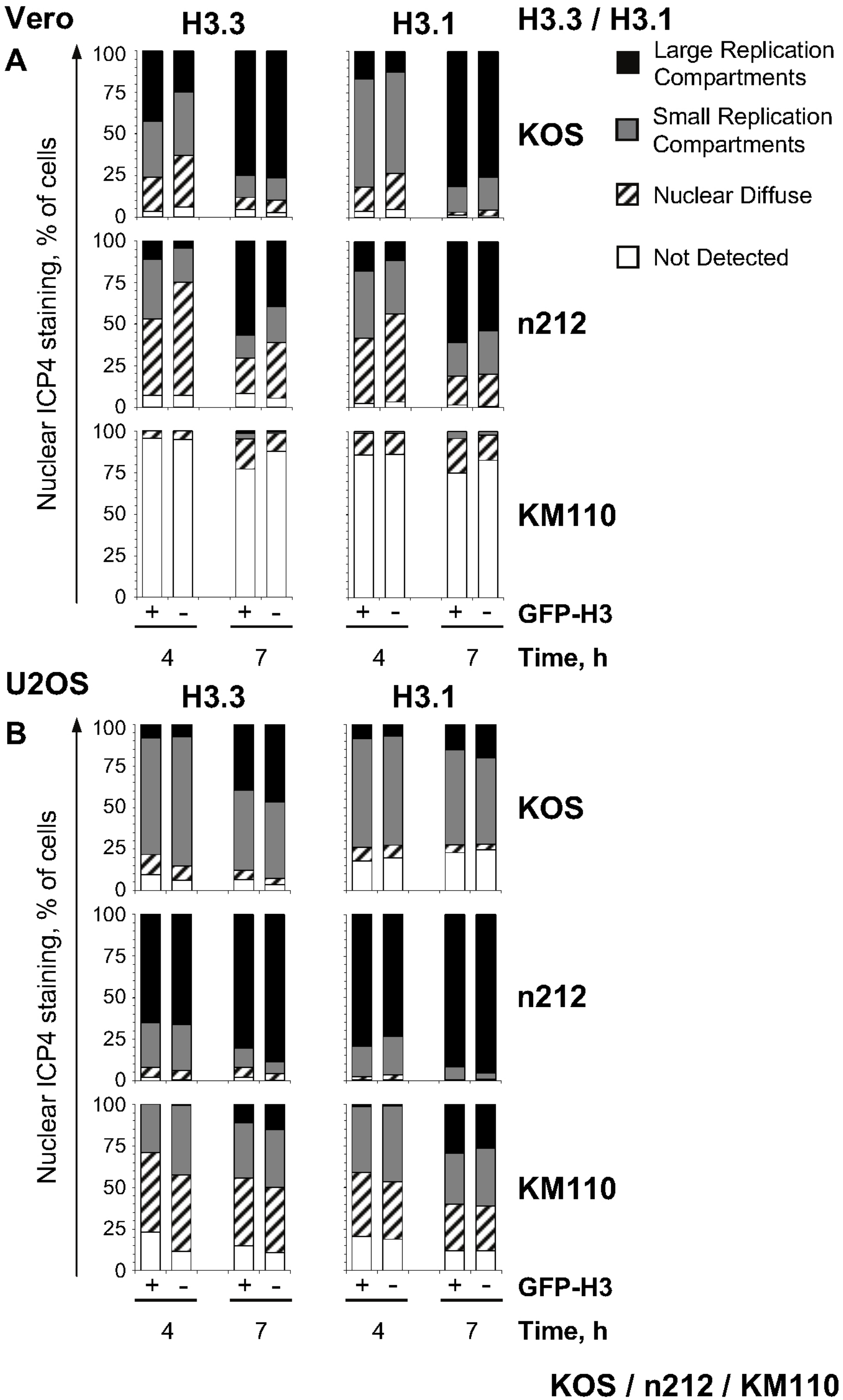

Vero
A
H3.3
H3.1
H3.3 / H3.1
Large Replication Compartments
Small Replication Compartments
Nuclear Diffuse
Not Detected
KOS
n212
KM110
Nuclear ICP4 staining, % of cells
0
25
50
75
100
+
-
GFP-H3
4
7
Time, h
U2OS
B
KOS / n212 / KM110
