# Supplementary figures and images for: The Differential Mobilization of Histones H3.1 and H3.3 by Herpes Simplex Virus 1 Relates Histone Dynamics to the Assembly of Viral Chromatin
Source: PLoS Pathog. 2013 Oct 10;9(10):e1003695. doi: 10.1371/journal.ppat.1003695 (PMC3795045; doi:10.1371/journal.ppat.1003695)

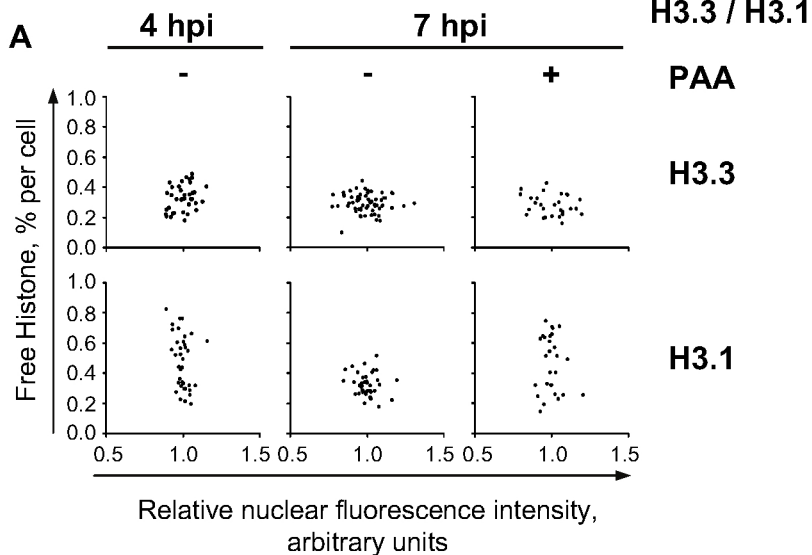**B****H3.3**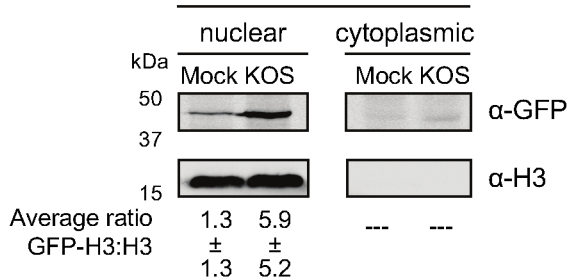**H3.1**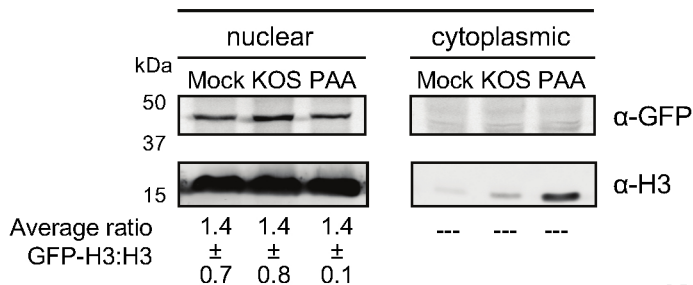**KOS**

Supplement: Figure S3 — Levels of free GFP-H3.3 or -H3.1 in infected cells do not correlate with expression levels. (A) Levels of free GFP-H3 per individual cell plotted against normalized fluorescence intensity. Vero cells were transfected with plasmids expressing GFP-H3.3 (H3.3) or -H3.1 (H3.1). At least 12 (H3.3) or 24 (H3.1) hours after transfection, cells were mock-infected or infected with 30 PFU/cell of strain KOS and treated (+) or not (−) with 400 µg PAA. Levels of free GFP-H3.3 or -H3.1 were evaluated 4 to 5 (4 hpi) or 7 to 8 (7 hpi) hours later by FRAP. Correlation coefficients, H3.3 r2 = 0.104, 0.002, or 0.054 at 4, 7, or 7 hpi with PAA treatment, respectively; H3.1 r2 = 0.096, 0.022, or 0.006 at 4, 7, or 7 hpi with PAA treatment, respectively. (B) Western blots showing the expression levels of GFP-H3.3 or -H3.1 fusion proteins and endogenous H3. Cells were harvested at 4 hpi, nuclear and cytoplasmic extracts were resolved by SDS-PAGE, and the levels of GFP or H3 expression were analyzed by Western Blot. The average ratio of GFP-H3 to endogenous H3 signal intensities calculated from 3 (H3.3), 4 (H3.1), or 2 (H3.1 with PAA) experiments is presented; dashes, ratio could not be calculated (at least one value is 0). (PDF) [file ppat.1003695.s003.pdf]

# U2OS

# H3.3 / H3.1

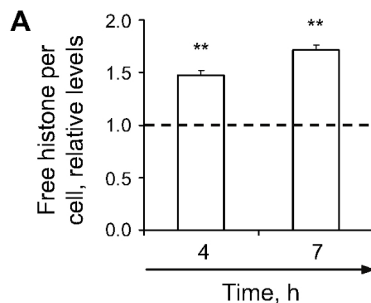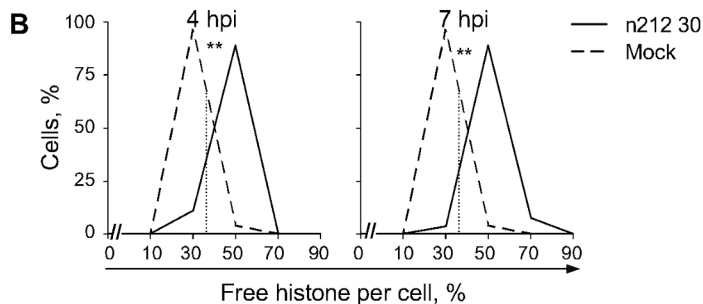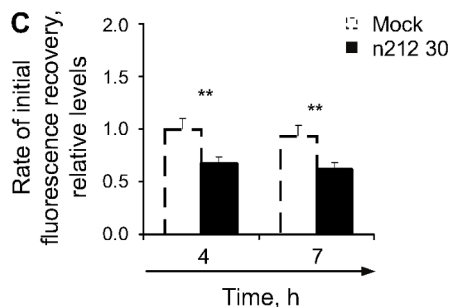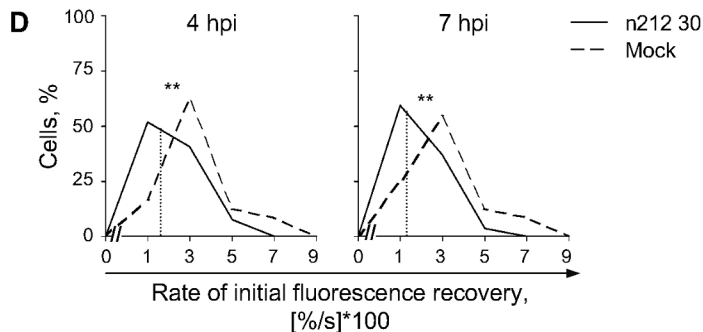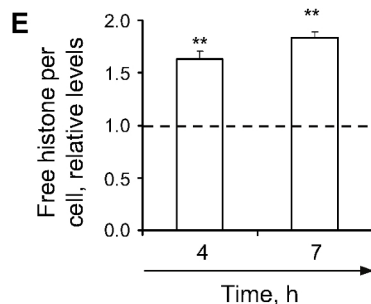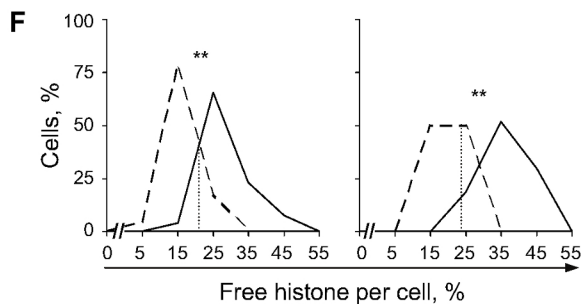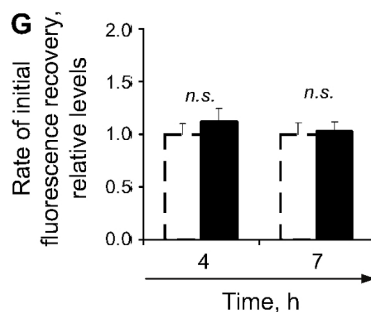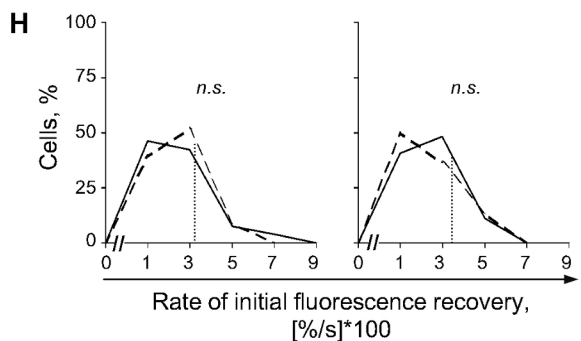

Supplement: Figure S4 — The degree of GFP-H3.1 or -H3.3 mobilization in U2OS cells correlates with infection progression. (A) Average normalized levels of free GFP-H3.1 relative to mock-infected cells at 4 or 7 hpi, respectively. U2OS cells were transfected with plasmids expressing GFP-H3.1 (H3.1) or -H3.3 (H3.3). Transfected cells were mock-infected or infected with 30 PFU/cell of strain n212. Mobilization of GFP-H3.1 or -H3.3 was examined from 4 to 5 (4) or 7 to 8 (7) hpi by FRAP; error bars, SEM; dashed line, normalized average level of free GFP-H3.1 in mock-infected cells. (B) Frequency distribution plots of the percentage of free GFP-H3.1 per individual cell at 4 or 7 hpi; dotted line, one SD above the average level of free GFP-H3.1 in mock-infected cells. (C) Average initial rate of normalized fluorescence recovery relative to mock-infected cells at 4 hpi; error bars, SEM. (D) Frequency distribution plots of the initial rate of normalized fluorescence recovery of GFP-H3.1 per individual cell; dotted line, one SD below the average initial rate of normalized fluorescence recovery in mock-infected cells. (E) Average normalized levels of free GFP-H3.3 relative to mock-infected cells at 4 or 7 hpi, respectively; error bars, SEM; dashed line, normalized average level of free GFP-H3.3 in mock-infected cells. (F) Frequency distribution plots of the percentage of free GFP-H3.3 per individual cell at 4 or 7 hpi; dotted line, one SD above the average level of free GFP-H3.1 in mock-infected cells. (G) Average initial rate of normalized fluorescence recovery relative to mock-infected cells at 4 hpi; error bars, SEM. (H) Frequency distribution plots of the initial rate of normalized fluorescence recovery of GFP-H3.3 per cell; dotted line, one SD above the average initial rate of normalized fluorescence recovery in mock-infected cells. **, P<0.01; n.s., not significant. (PDF) [file ppat.1003695.s004.pdf]
