# Supplementary material for: The Differential Mobilization of Histones H3.1 and H3.3 by Herpes Simplex Virus 1 Relates Histone Dynamics to the Assembly of Viral Chromatin
Source: PLoS Pathog. 2013 Oct 10;9(10):e1003695. doi: 10.1371/journal.ppat.1003695 (PMC3795045; doi:10.1371/journal.ppat.1003695)
Supplement: Methods S1 — Supplementary materials and methods information. (DOCX) [file ppat.1003695.s005.docx]

**SUPPLEMENTAL MATERIALS AND METHODS**

**Immunofluorescence.** Cells were transfected and infected as described in Materials and Methods. Immunofluorescence was performed as described [25] in cells permeabilized with -20°C MeOH. α-ICP4 mouse monoclonal (1101-897, Goodwin Institute for Cancer Research Inc, Plantation, Florida; or ab6514, Abcam) was diluted 1:15,000 in blocking buffer and incubated for 2h with slow rocking. AlexaFluor 594-labelled goat α-mouse (Molecular probes) was diluted 1:1,000 in blocking buffer and incubated for 1h with slow rocking. Nuclei were counterstained with 1 μg/ml Hoechst 33258. Coverslips were mounted with Vectashield mounting medium (Vector) and sealed with clear nail enamel, or with Mowiol (G. Barron; Cross Cancer Institute, Edmonton, AB). The cells were viewed on a Leica DM IRB microscope. A minimum of 200 transfected or non-transfected cells from at least three experiments were counted at 4 and 7hpi, except for Vero H3.3 transfected cells infected with KM110 or n212 at 4 and 7hpi, in which a minimum of 100 cells from 3 experiments were counted, and Vero H3.1 transfected cells infected with KM110 at 7hpi in which 120 cells from 1 experiment were counted.

**Nuclear and cytoplasmic lysate preparation.** Cells were transfected and infected as described in Materials and Methods. Cells were trypsinized at 4hpi and collected in 37°C DMEM supplemented with 5% FBS. Cells were lysed in hypotonic RSB buffer (10mM Tris (pH 7.5), 10mM NaCl, 5mM MgCl_2_) with 0.5% (v/v) Nonidet P-40 (NP-40). Nuclei were isolated by centrifugation (1,811 x g, for 25min at 4°C), and lysed and in NP-40 lysis Buffer (50mM Tris (pH 7.6), 150mM NaCl, 1% NP-40) containing benzonase (25U per sample; Novagen) to digest the nucleic acids. Proteins from the cytoplasmic and nuclear lysates were precipitated with -20°C acetone at -20°C, and pelleted by centrifugation at 14,000 x g for 10min at 4°C. Protein pellets were resuspended in 10mM Tris (pH 7.5), and re-precipitated with -20°C acetone at -20°C. Proteins were pelleted by centrifugation at 14,000 x g for 10min at 4°C.

**Western blots.** Nuclear and cytoplasmic proteins were resolved in 12% SDS-PAGE gels (Mini-PROTEAN; Bio-Rad Laboratories). Proteins were transferred to polyvinylidene di-fluoride (PVDF) membranes (Immuno-Blot, 0.2μM; Bio-Rad Laboratories) by wet transfer in 49.6mM Tris, 384mM glycine, and 20% methanol for 1h at 1mA/cm^2^, 15h at 3.5mA/cm^2^, and 2h at 7mA/cm^2^ at 6°C. Temperature was maintained by heat exchange (Isotemp 1016D; Thermo Fisher Scientific, Waltham, Massachusetts, USA). All subsequent procedures were performed at room temperature and with gentle rocking unless otherwise indicated. Membranes were blocked for 1h in 10% blocking buffer (Sigma-Aldrich), then probed for 18h at 4°C with rabbit polyclonal anti-GFP antibodies (Dr. L. Berthiaume; University of Alberta) diluted 1:20,000 in 10% blocking buffer and 0.1% Tween-20. Following one 5min and three 10min washes in PBS with 0.1% Tween-20, membranes were probed with goat anti-rabbit IRDye 800-labelled secondary antibodies (LI-COR Biosciences) diluted 1:20,000 in 10% blocking buffer with 0.1% Tween-20 and 0.01% SDS for 1h. Following one 5min and three 10min washes in PBS with 0.1% Tween-20, signal from the IRDye 800-labeled secondary antibody was detected at 800nm and that of the pre-stained proteins standards at 700nm using an Odyssey infrared imaging system. Signal was quantitated using Odyssey 3.0 software.

Membranes were stripped in 25mM glycine, 1% SDS, pH 2.0 once for 5min and thrice for 10min, rinsed once for 5min in PBS with 0.1% Tween-20, and rinsed once for 5min in PBS. Following incubation for 1h in 10% blocking buffer, membranes were probed with rabbit polyclonal anti-H3 antibodies (ab1791; Abcam) diluted 1:5,000 in 10% blocking buffer and 0.1% Tween-20 as described above.

**Mitotic cell imaging.** Cells were transfected as described in Materials and Methods. Transfected cells were seeded on glass bottom tissue culture dishes. At least 4h after seeding, live cells were imaged using a spinning disk confocal system (PerkinElmer Ultraview ERS) with a Zeiss Axiovert 200M inverted microscope. Cells were viewed on a Plan-Neofluar 40x oil immersion objective lens (NA 1.3) heated to 37°C in 5% CO_2_.

covery in mock-infected cells. **, P<0.01; *n.s.*, not significant.
