# Supplementary figures and images for: Correction:The Differential Mobilization of Histones H3.1 and H3.3 by Herpes Simplex Virus 1 Relates Histone Dynamics to the Assembly of Viral Chromatin
Source: PLoS Pathog. 2016 Apr 13;12(4):e1005575. doi: 10.1371/journal.ppat.1005575 (PMC4830543; doi:10.1371/journal.ppat.1005575)

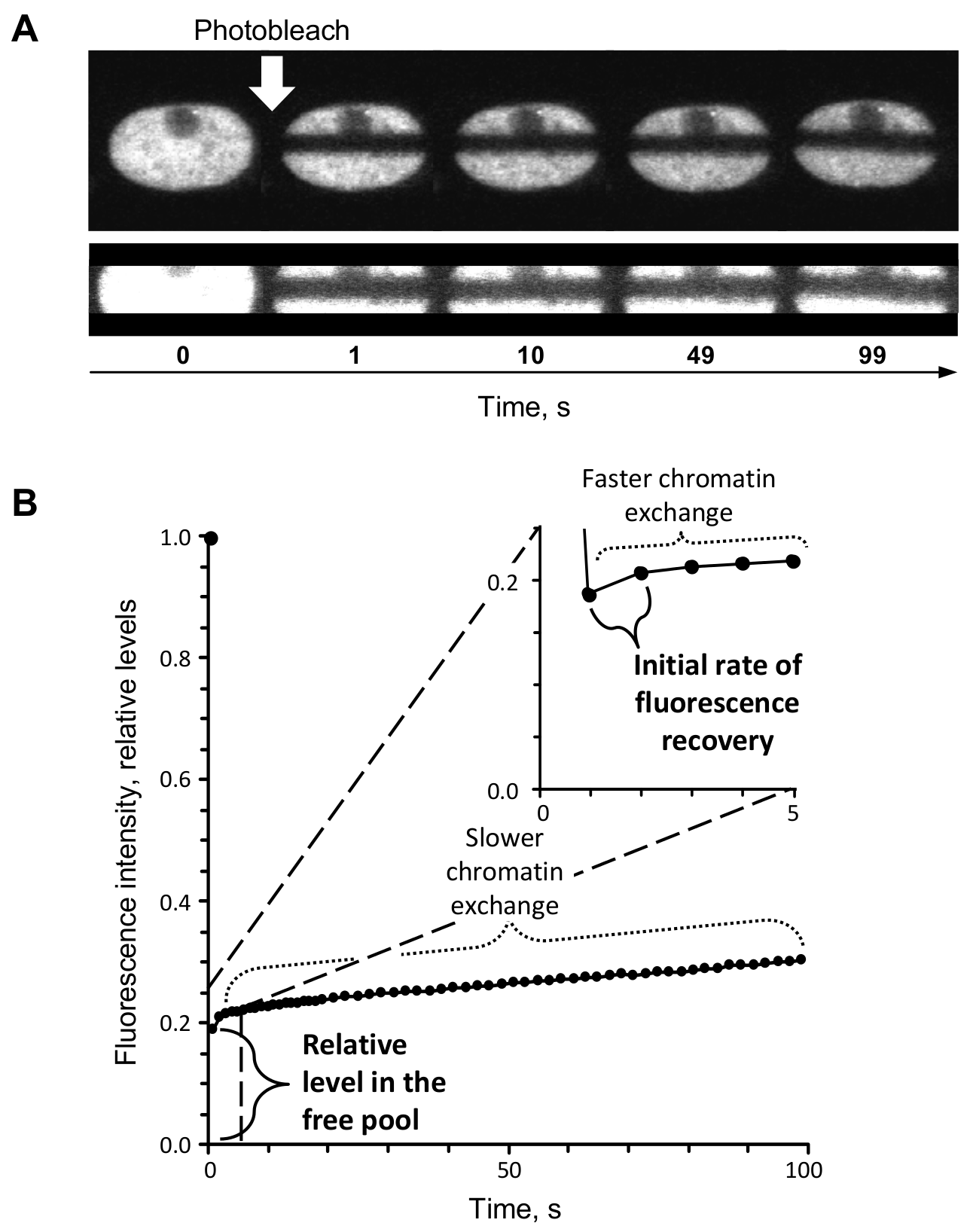

Supplement: S1 File — (TIF) [file ppat.1005575.s001.tif]
